# Supplementary material for: Analysis of PPARγ Signaling Activity in Psoriasis
Source: Int J Mol Sci. 2021 Aug 10;22(16):8603. doi: 10.3390/ijms22168603 (PMC8395241; doi:10.3390/ijms22168603)
Supplement: Supplementary file 1 [file ijms-22-08603-s001.zip › Supplemental materials_Analysis of PPARg signaling activity in psoriasis/Pathway models/Models images and html files/Differentiation of psoriatic T cells/11524.html]

TNF


# Protein TNF

|  |  |
| --- | --- |
| URN | urn:agi-llid:7124 |
| Total Entities | 0 |
| Connectivity | 30357 |
| Name | TNF |
| Description | tumor necrosis factor |
| Notes | This gene encodes a multifunctional proinflammatory cytokine that belongs to the tumor necrosis factor (TNF) superfamily. This cytokine is mainly secreted by macrophages. It can bind to, and thus functions through its receptors TNFRSF1A/TNFR1 and TNFRSF1B/TNFBR. This cytokine is involved in the regulation of a wide spectrum of biological processes including cell proliferation, differentiation, apoptosis, lipid metabolism, and coagulation. This cytokine has been implicated in a variety of diseases, including autoimmune diseases, insulin resistance, and cancer. Knockout studies in mice also suggested the neuroprotective function of this cytokine. [provided by RefSeq, Jul 2008] |
| Primary Cell Localization | Extracellular |
| Class | Ligand |
| ObjectType | Protein |

---

|  |  |
| --- | --- |
| Pathway | Longevity Related Drugs |
|  | Proteins with Altered Expression in Aging |
|  | Alzheimer's Disease |
|  | Osteoarthritis |
|  | Skin Aging |
|  | Androgen Deficiency in Male Obesity |
|  | MTHFR Activity Regulation |
|  | mTOR Signaling Hyperfunction |
|  | Prolactin Production |
|  | Somatostatin Receptors Signaling |
|  | Cortisol in Resolving Inflammation |
|  | Toll-like Receptors Act through MYD88-TIRAP Signaling |
|  | Antiviral Signaling through Pattern Recognition Receptors |
|  | DCIR1 (CLEC4A) Signaling |
|  | TLR4 Signaling in Leukocytes |
|  | Dectin-2 (CLEC6A)/Mincle (CLEC4E)/BDCA2 (CLEC4C) Signaling |
|  | Natural Killer Cell Activation through C-Type Lectin-like Receptors |
|  | Natural Killer Cell Activation through ITAM-Containing Receptors |
|  | Natural Killer Cell Activation through ITSM-Containing Receptors |
|  | T-Cell Maturation (Hypothesis) |
|  | Peripheral T-Cell Tolerance |
|  | Plasmin Effects in Inflammation |
|  | Platelet Activation via Adhesion Molecules |
|  | Proplatelet Maturation |
|  | Toll-like Receptors in Sterile Inflammation |
|  | Toll-like Receptor Independent Sterile Inflammation |
|  | Necrosis |
|  | Hypothalamic-Pituitary-Adrenal Axis in Acute Phase Response |
|  | MC1R in anti-Inflammatory Signaling |
|  | Lipoxins and Resolvins in Inflammation Resolution |
|  | Lipids Enhance Apoptotic Cell Engulfment and Reduce Inflammation |
|  | Keep out Signal: LTF Inhibits Neutrophil Recruitment and Inflammation |
|  | Leukotriene Effect on Vascular Endothelial Cell Response |
|  | Basophil Activation |
|  | Eosinophil Survival by Cytokine Signaling |
|  | Eosinophil Activation |
|  | Mast-Cell Activation via IgE Signaling |
|  | Mast-Cell Activation without Degranulation |
|  | Mast-Cells De Novo Synthesized Mediators via IgE Independent Signaling |
|  | Mast-Cell Activation without Degranulation through IL1R1 and TLR Signaling |
|  | Macrophage Related Iron Uptake and Release |
|  | Macrophage M1 Lineage |
|  | CD16/CD14 Proinflammatory Monocyte Activation |
|  | AHR Signaling in M1 Macrophages Function |
|  | anti-Inflammatory Function of Macrophage M2 Lineage |
|  | AXL Receptor Inhibits Macrophages and Dendritic Cells Function |
|  | Neutrophil Recruitment and Priming |
|  | Negative Acute Phase Proteins Synthesis |
|  | Positive Acute Phase Proteins Synthesis |
|  | Vascular Endothelial Cell Activation by Cytokines |
|  | Vascular Endothelial Cell Activation by Blood Coagulation Factors |
|  | Adipokines Production by Adipocyte |
|  | Lipolysis Regulation in Adipocyte |
|  | GFs/TNF -> Ion Channels |
|  | Nociception Expression Targets Signaling |
|  | CCR1 Expression Targets |
|  | HTR5 -> TNF Production |
|  | TACR1 -> TNF/IL6/IL8 Production |
|  | IL1B Expression Targets -> Nociception |
|  | Monoblast -> Promonocyte Surface Expression Markers |
|  | Apoptosis |
|  | Proteins Involved in Atherosclerosis |
|  | Dendritic Cells Function in Atherosclerosis |
|  | Dilated Cardiomyopathy |
|  | Proteins Involved in Dilated Cardiomyopathy |
|  | Proteins Involved in Hypertrophic Cardiomyopathy |
|  | Cardiomyocyte Hypertrophy |
|  | Hypertrophic Cardiomyopathy |
|  | Proteins Involved in Arterial Hypertension |
|  | Proteins Involved in Pulmonary Hypertension |
|  | Myocardial Infarction |
|  | Proteins Involved in Myocardial Ischemia |
|  | Proteins Involved in Myocarditis |
|  | Apoptosis of Myocyte |
|  | SOCS in Negative Feedback Regulation in Myocarditis |
|  | Toll-like Receptors (TLR) in Antiviral Innate Immune Response |
|  | Proteins Involved in Vasospasm |
|  | Proteins Involved in Acne Vulgaris |
|  | Inflammatory Reaction in Acne Vulgaris |
|  | Hair Follicle Keratinocyte Apoptosis |
|  | Mast-Cells Activation in Atopic Dermatitis |
|  | Proteins Involved in Atopic Dermatitis |
|  | Proteins Involved in Psoriasis |
|  | Genes with Mutations Associated with Psoriasis |
|  | T-Cells Differentiation Block in Psoriasis |
|  | IFN-gamma/TNF-alpha Mediated Cell Proliferation in Psoriasis |
|  | Dendritic Cells Function in Psoriasis |
|  | Proteins Involved in Photoaging |
|  | Dendritic Cell Activation in Systemic Lupus Erythematosis |
|  | Th0-Cell Aberrant Activation in Systemic Lupus Erythematosus |
|  | Proteins Involved in Systemic Lupus Erythematosus |
|  | Neutrophil and Macrophage Function in Systemic Lupus Erythematosus |
|  | Proteins Involved in Vitiligo |
|  | Oxidative Stress in Vitiligo |
|  | Genes with Mutations Associated with Vitiligo |
|  | Vitiligo |
|  | Plasmacytoid Dendritic Cell in Diabetes Mellitus Type 1 |
|  | Toll-like Receptors in beta-Cell |
|  | Macrophage Function in Diabetes Mellitus Type 1 |
|  | Proteins Involved in Diabetes Mellitus Type 1 |
|  | Conventional Dendritic Cell Role in Diabetes Mellitus Type 1 |
|  | Proteins Involved in Diabetes Mellitus Type 2 |
|  | Podocyte Dysfunction in Diabetic Nephropathy |
|  | Proteins Involved in Diabetic Nephropathy |
|  | Mesangial Cell Dysfunction in Diabetic Nephropathy |
|  | Proteins Involved in Diabetic Neuropathy |
|  | Adipokines and Cytokines in Insulin Resistance in Skeletal Muscles |
|  | Proteins Involved in Insulin Resistance |
|  | Adipose Tissue Inflammation Provokes Insulin Resistance |
|  | Adiponectin Synthesis Declines in Insulin Resistance |
|  | Insulin Resistance in Hepatocytes |
|  | Hepatic Stellate Cells in non-Alcoholic Fatty Liver Disease |
|  | Proteins Involved in non-Alcoholic Fatty Liver Disease |
|  | Oxidation of Fatty Acid in non-Alcoholic Fatty Liver Disease |
|  | beta-Cell Destruction through Cytokines in Diabetes Mellitus |
|  | Proteins Involved in Hashimoto's Thyroiditis |
|  | Hashimoto's Thyroiditis |
|  | Apoptosis of Thyroid Cell in Hashimoto's Thyroiditis |
|  | Proteins Involved in Graves Disease |
|  | Proteins Involved in Hypothyroidism |
|  | Thymic Follicular Hyperplasia |
|  | Fatty Acids in Hypertension and Atherosclerosis Development |
|  | Adipokines Production by Adipocyte Impaired in Obesity |
|  | Proteins Involved in Obesity |
|  | Proteins Involved in Celiac Disease |
|  | Proteins Involved in Colorectal Neoplasms |
|  | Crohn's Disease |
|  | Macrophage Dysfunction |
|  | Proteins Involved in Inflammatory Bowel Diseases |
|  | Dendritic Cell Dysfunction in Crohn's disease |
|  | Proteins Involved in Helicobacter Infections |
|  | Dendritic Cell Function in Ulcerative Colitis |
|  | Epithelial Cell in the Innate Immune Response in Ulcerative Colitis |
|  | Nicotine anti-Inflammatory Effect in Ulcerative Colitis |
|  | Proteins Involved in Ulcerative Colitis |
|  | Proteins Involved in Antiphospholipid Syndrome |
|  | Proteins Involved in B-Cell Chronic Lymphocytic Leukemia |
|  | Hodgkin and Reed-Sternberg Cells in Hodgkin Lymphoma |
|  | Proteins Involved in Hodgkin Lymphoma |
|  | Hodgkin Lymphoma |
|  | Mantle Cell Lymphoma |
|  | mTOR/NF-kB/BCR Signaling Disregulation in Mantle Cell Lymphoma |
|  | Splenic Marginal Zone Lymphoma |
|  | TNFR/NF-kB Classical Pathway in Multiple Myeloma Plasma Cells |
|  | IL6/IGF1/VEGFA Signaling in Multiple Myeloma |
|  | Multiple Myeloma |
|  | Proteins Involved in Multiple Myeloma |
|  | TNFR/NF-kB Alternative Pathway in Multiple Myeloma Plasma Cells |
|  | Proteins Involved in Thrombophilia |
|  | Proteins Involved in alpha 1-Antitrypsin Deficiency |
|  | Proteins Involved in Hepatocellular Carcinoma |
|  | Proteins Involved in Pancreatic Cancer |
|  | TRPM7/8 in Pancreatic Adenocarcinoma |
|  | Goblet-Cell Related Mucus Secretion in Asthma |
|  | Basophil Activation in Asthma |
|  | Antigen-Presenting Cell Role in Asthma |
|  | CFTR Facilitates Oxidative Stress in Airway Epithelium in CF |
|  | Proteins Involved in Cystic Fibrosis |
|  | Airway Epithelial Cell Dysfunction |
|  | CFTR Related Ion-Channel Dysfunction in Cystic Fibrosis Airway Epithelium (Class III Mutations) |
|  | Proteins Involved in Gout |
|  | Neutrophil Recruitment in Sinovium in Gout |
|  | Monocytes Function in Gout |
|  | TNF and IL1B Induce Metalloproteinase Synthesis in Osteoarthritis |
|  | Apoptosis of Chondrocyte |
|  | Proteins Involved in Osteoarthritis |
|  | Proteins Involved in Polycystic Ovary Syndrome |
|  | Dendritic Cell Dysfunction in Psoriatic Arthritis |
|  | Synovial Fibroblast Activation in Psoriatic Arthritis |
|  | Keratinocyte Activation in Psoriatic Arthritis |
|  | Proteins Involved in Psoriatic Arthritis |
|  | Synovial Fibroblast Proliferation in Rheumatoid Arthritis |
|  | Synovial Fibroblast Activation by Citokines in Rheumatoid Arthritis |
|  | Apoptosis Block in Synovial Fibroblast |
|  | TLR2 Induced Synovial Fibroblast Activation in Rheumatoid Arthritis |
|  | Proteins Involved in Rheumatoid Arthritis |
|  | FAS Mediated Apoptosis in Systemic Scleroderma |
|  | Genes with Mutations Associated with Systemic Scleroderma |
|  | Proteins Involved in Systemic Scleroderma |
|  | Skin Fibrosis |
|  | Proteins Involved in Dental Caries |
|  | Dentin and Enamel Dysruprion in Bloch-Sulzberger Syndrome |
|  | Proteins Involved in HIV Type 1 Infection |
|  | Apoptosis Block in Infected Cells |
|  | Toll-like Receptors (TLR) in Periodontitis |
|  | Diabetes Induced Periodontitis |
|  | Proteins Involved in Periodontitis |
|  | IL1 Receptor Antagonist (IL1RN) Deficiency (DIRA) |
|  | PAPA Syndrome |
|  | TNF Receptor Associated Periodic Syndrome |
|  | Mevalonate Kinase Deficiency |
|  | IL36 Receptor Antagonist (IL1F5) Deficiency (DITRA) |
|  | Cryopyrin (NLPR3) Associated Periodic Syndromes |
|  | Familial Cold Autoinflammatory Syndrome 2 |
|  | Familial Mediterranean Fever |
|  | Majeed Syndrome |
|  | NF-kB Signaling Activation in Huntington Disease |
|  | Proteins Involved in Retinitis Pigmentosa |
|  | Oxytocin Signaling in Brain Nerve Cell (Hypothesis) |
|  | Gluten Impact on Neuronal System (Hypothesis) |
|  | beta-Casomorphins/Opioid Receptors Affect Brain Function (Hypothesis) |
|  | Muscular Dystrophies |
|  | Proteins with Altered Expression in Osteoarthritis |
|  | Proteins Involved in Osteoporosis |
|  | Bone Loss in Osteoporosis |
|  | Proteins with Altered Expression in Osteoporosis |
|  | Osteoclast Activation in Postmenopause |
|  | Bone Resorption by Osteoclasts in Osteoporosis |
|  | Genes with Mutations Associated with Psoriatic Arthritis |
|  | Proteins with Altered Expression in Psoriatic Arthritis |
|  | Proteins with Altered Expression in Rheumatoid Arthritis |
|  | Microglia Activation in Alzheimer's Disease |
|  | Proteins Involved in Alzheimer's Disease |
|  | Proteins Involved in Epilepsy |
|  | Ketogenic Diet Mechanism (Hypothesis) |
|  | HMGB1 and IL1B in Neuroinflammation |
|  | Apoptosis and Immediate Early Gene Activation in Epileptiform Disorders |
|  | Proteins Involved in Glioblastoma |
|  | Proteins Involved in Astrocytoma |
|  | Proteins Involved in Glioma |
|  | Proteins Involved in Migraine |
|  | Proteins Involved in Neuroblastoma |
|  | Microglia Activation in Parkinson's Disease |
|  | Proteins with Altered Expression in Parkinson's Disease |
|  | Proteins Involved in Parkinson's Disease |
|  | Proteins with Altered Expression in Amyotrophic Lateral Sclerosis |
|  | Proteins Involved in Amyotrophic Lateral Sclerosis |
|  | Microglia and Motor Neuron Interaction Dysregulation |
|  | Neuroinflammation in Amyotrophic Lateral Sclerosis |
|  | Crystallin alpha B (CRYAB) Suppresses Multiple Sclerosis |
|  | MBP/MOG/PLP in Immune System Activation |
|  | Proteins Involved in Multiple Sclerosis |
|  | Proteins Involved in Sleep Disorders |
|  | Proteins Involved in Vascular Dementia |
|  | Proteins Involved in Adhesion, Migration and Invasion of Endometriotic Cell |
|  | Proteins Involved in Endometriosis |
|  | Proteins with Altered Expression in Endometriosis |
|  | Coagulation Factors and PAR1/2 Receptors (F2R/F2RL1) in Endometriosis |
|  | Leptin Role in Endometriosis |
|  | Proteins Involved in Spontaneous Abortion |
|  | Proteins Involved in Cholesteatoma |
|  | Subepithelial Fibroblasts in Middle Ear Cholesteatoma |
|  | HPV Infection and Cancer |
|  | HPV E6 and E7 Induced Disruption of TNFR/NF-kB Signaling in Keratinocytes |
|  | Androgen Receptor/NKX3-1 Signaling |
|  | Androgen Receptor/Protein Kinase C-delta Signaling |
|  | Proteins Involved in Age-Related Macular Degeneration |
|  | OPTN Effects on NF-kB Signaling in Glaucoma |
|  | Proteins Involved in Glaucoma |
|  | Glutamate Mediated Excitotoxicity in Glaucoma |
|  | Proteins Involved in Noise-Induced Hearing Loss |
|  | Proteins Involved in Presbycusis |
|  | Aminoglycosides and Cisplatin Induced Ototoxicity (Mouse Model) |
|  | Proteins Involved in Hearing Loss |
|  | Cochlear Inflammation (Noise-Induced) |
|  | Proteins Involved in Otitis Media |
|  | Cytokines Trigger Otitis Media |
|  | Immune System Role in Otitis Media |
|  | Genes with Mutations Associated with Asthma |
|  | Alveolar Macrophage Negative Regulation Declined in COPD |
|  | Alveolar Epithelial Cell Apoptosis in COPD |
|  | Alveolar Macrophages Dysfunction in COPD |
|  | Glucocorticoid Resistance |
|  | Proteins Involved in Chronic Obstructive Pulmonary Disease |
|  | T-Cell Cytotoxic Activation |
|  | Alveolar Epithelial Cell Dysfunction in COPD |
|  | Proteins Involved in Pulmonary Emphysema |
|  | Mucin Hyperproduction in Goblet and Mucous Cells |
|  | Proteins Involved in Chronic Bronchitis |
|  | Proteins Involved in Polycystic Kidney Disease |
|  | Proteins Involved in Erectile Dysfunction |
|  | Proteins Involved in Glomerulonephritis |
|  | Proteins with Altered Expression in Glomerulonephritis |
|  | Plasmin Role in Glomerulonephritis |
|  | Endothelial Cell Dysfunction in Glomerulonephritis |
|  | Podocyte Dysfunction in Glomerulonephritis |
|  | Mesangial Cell Dysfunction in Glomerulonephritis |
|  | Interstitial Fibroblasts in Pyelonephritis |
|  | Apoptosis of Renal Tubule Epithelial Cell in Pyelonephritis |
|  | Proteins Involved in Pyelonephritis |
|  | Proteins with Altered Expression in Pyelonephritis |
|  | Toll-like Receptors in Pyelonephritis |
|  | Endothelial Cell Dysfunction in Pyelonephritis |
|  | HMGB1 Expression Targets |
|  | S100B Expression Targets |
|  | Noradrenaline/Gs Expression Targets |
|  | Dopamine/Gs Expression Targets |
|  | Epinephrine/Gs Expression Targets |
|  | Acetylcholine Expression Targets |
|  | Dopamine/Gi Expression Targets |
|  | Elevated Receptors -> Expression Targets in Adipose Tissue |
|  | GPCR Family -> Expression Targets in Adipose Tissue |
|  | GPCRs Family -> Expression Targets in Bone |
|  | CD2 Expression Targets |
|  | FCGR3A Expression Targets |
|  | CD19/CR2 Expression Targets |
|  | CD8 -> ATF/CREB/CREBBP Expression Targets |
|  | CD8 -> NF-kB Expression Targets |
|  | CD8 -> NFATC Expression Targets |
|  | CD8 -> STAT Expression Targets |
|  | CD80 -> AP-1 Expression Targets |
|  | CTF1 Expression Targets |
|  | CXCL12 Expression Targets |
|  | CCL16 Expression Targets |
|  | CCL5 Expression Targets |
|  | CCL2 Expression Targets |
|  | CSF2 -> NF-kB Expression Targets |
|  | CSF2 -> STAT Expression Targets |
|  | EGF -> CTNN Expression Targets |
|  | EGF -> AP-1/ATF Expression Targets |
|  | EGF -> CREB/CREBBP/ELK/SRF/MYC Expression Targets |
|  | EGF -> MEF/MYOD/NFATC Expression Targets |
|  | EGF -> STAT Expression Targets |
|  | Erythropoietin -> AP-1/MYC/CREB Expression Targets |
|  | F2 -> AP-1/CREB/ELK/SRF/SP1 Expression Targets |
|  | F2 -> STAT1/NF-kB Expression Targets |
|  | PLG -> AP-1/CREB/ELK/SRF/SP1 Expression Targets |
|  | PLG -> STAT1/NF-kB Expression Targets |
|  | FGF7 -> AP-1/CREB/CREBBP/MYC Expression Targets |
|  | GCG Expression Targets |
|  | ADCYAP1 Expression Targets |
|  | FSHR Expression Targets |
|  | Glutamate/Gq Expression Targets |
|  | VIP Expression Targets |
|  | GH1/GHR -> NF-kB/ELK/SRF/MYC Expression Targets |
|  | GH1/GHR -> STAT Expression Targets |
|  | GH1/PRLR Expression Targets |
|  | NFKB Canonical Signaling Activation in Cancer |
|  | NFKB Non-Canonical Signaling Activation in Cancer |
|  | STAT3 and NFkB Activate Inflammation-Induced Tumorigenesis |
|  | IL23A and IL17A Provoke Cancer-Associated Inflammation |
|  | Asbestosis- and Silicosis-Associated Inflammation Provokes Cancer Development |
|  | Proteins with Altered Expression in Tumor-Promoting Inflammation |
|  | NFKB Signaling Activation by Blocking of Tumor Suppressors |
|  | Apoptosis Block by Death Receptor Signaling in Cancer |
|  | Apoptosis Evasion in Cancer: Overview |
|  | VEGFA Dependent Angiogenesis in Cancer |
|  | Cancer Cells Inhibit Adipocyte Differentiation |
|  | Skeletal Muscle Wasting in Cancer Cachexia |
|  | Proteins with Altered Expression in Cancer Metabolic Reprogramming |
|  | IDO1 in Cancer Immune Escape |
|  | Proteins with Altered Expression in Cancer Immune Escape |
|  | Effector T-cell Inactivation in Cancer Immune Escape |
|  | HCAR1/HCAR2 -> MAPK Signaling |
|  | Fibronectin Expression Targets |
|  | IFNG/IFNR Expression Targets |
|  | IL7 Expression Targets |
|  | IL13 Expression Targets |
|  | IL21 Expression Targets |
|  | IL15 Expression Targets |
|  | IL16 -> AP-1 Expression Targets |
|  | IL3 Expression Targets |
|  | IL4 Expression Targets |
|  | IL2 Expression Targets |
|  | Leptin -> STAT Expression Targets |
|  | Leptin -> ELK/SRF Expression Targets |
|  | Thromboxane A2 Expression Targets |
|  | PAF/Gq -> AP-1/ATF1/CREB/ERK/SRF Expression Targets |
|  | Dronabinol/Anandamide Expression Targets |
|  | Morphine Expression Targets |
|  | PAF/Gq -> NF-kB Expression Targets |
|  | S1P Expression Targets |
|  | Proteins/Ions Transporters -> Expression Targets in Lymphoid System and Blood |
|  | Elevated Receptors -> Expression Targets in Lymph Node |
|  | Elevated Receptors -> Expression Targets in Lymphoid System and Blood |
|  | CD Markers -> Expression Targets in Lymphoid System and Blood |
|  | GPCRs Family -> Expression Targets in Lymphoid System and Blood |
|  | TNF Receptors -> Expression Targets in Lymphoid System and Blood |
|  | Interleukin Receptors -> Expression Targets in Lymphoid System and Blood |
|  | CD40 -> Expression Targets in Thymus |
|  | C5AR1 -> Expression Targets in Lymphoid System and Blood |
|  | IgE Receptors -> Targets in Lymphoid System and Blood |
|  | Elevated Receptors -> Expression Targets in Thymus |
|  | P2RX7 -> Expression Targets in Nerve Tissue |
|  | Elevated Receptors -> Expression Targets in Nerve Tissue |
|  | NGF -> SMAD3/NF-kB Expression Targets |
|  | NGF -> AP-1/TP53/MYC Expression Targets |
|  | NGF -> CREB/CEBPB/MEF2A Expression Targets |
|  | ADAM17 Expression Targets |
|  | Adenosine Expression Targets |
|  | NTS Expression Targets |
|  | POMC Expression Targets |
|  | AGT -> CREB Expression Targets |
|  | IFNA1/Gq Expression Targets |
|  | TAC1 Expression Targets |
|  | EDN3 Expression Targets |
|  | AGT -> STAT Expression Targets |
|  | PRL/PRLR Expression Targets |
|  | PRL/GHR -> STAT Expression Targets |
|  | PRL/GHR -> NF/kB/ELK/SRF/MYC Expression Targets |
|  | GAS6 -> AP-1/CREB Expression Targets |
|  | KITLG -> STAT Expression Targets |
|  | KITLG -> AP-1/CREB/CREBBP/MYC Expression Targets |
|  | Collagen -> NF-kB Expression Targets |
|  | PAF Expression Targets |
|  | PGF -> AP-1/CREB/CREBBP/MYC Expression Targets |
|  | Elevated Receptors -> Expression Targets in Lung |
|  | TNF-alpha/TNFRSF1A Signaling |
|  | MERTK Signaling |
|  | Ras-GAP Regulation Signaling |
|  | AXL Receptor Tyrosine Kinase Signaling |
|  | NF-kB non-Canonical Signaling |
|  | TNF-alpha/TNFRSF1B Signaling |
|  | NF-kB Canonical Signaling |
|  | TCR -> CREB/CREBBP/ATF Expression Targets |
|  | TCR -> NFAT Expression Targets |
|  | TCR -> AP-1 Expression Targets |
|  | TGFA -> STAT Expression Targets |
|  | TGFA -> FOXO3A Expression Targets |
|  | TGFA -> CTNNB/CTNND Expression Targets |
|  | TGFA -> MEF/MYOD/NFATC Expression Targets |
|  | TGFA/AP-1/ATF Expression Targets |
|  | TGFA -> CREB/CREBBP/ELK-SRF/MYC Expression Targets |
|  | TGFB2-TGFBR1 Expression Targets |
|  | TGFB2-TGFBR2 Expression Targets |
|  | TGFB1-TGFBR2 Expression Targets |
|  | TGFB1-ACVRL1 Expression Targets |
|  | TNF -> NF-kB Expression Targets |
|  | LTA Expression Targets |
|  | TNFSF14 Expression Targets |
|  | TNF -> AP-1 Expression Targets |
|  | CD72 -> AP-1 Expression Targets |
|  | CD40LG -> STAT Expression Targets |
|  | CD40LG -> NF-kB/ELK/SRF -> CREB/NFATC Expression Targets |
|  | TNF -> ELK-SRF Expression Targets |
|  | TNF -> CREB Expression Targets |
|  | TNF -> STAT Expression Targets |
|  | TNF -> TP53/ATF Expression Targets |
|  | TNFRSF1A -> STAT Signaling |
|  | TNFR -> CREB/ELK-SRF Signaling |
|  | TNFR -> AP-1/ATF/TP53 Signaling |
|  | TNFR -> NF-kB Signaling |
|  | TNFRSF1A -> AP-1/ATF/TP53 Signaling |
|  | TNFRSF1A -> CREB/ELK-SRF Signaling |
|  | TLR3 Expression Targets |
|  | TLR4 -> AP-1 Expression Targets |
|  | TLR5 Expression Targets |
|  | TLR7 Expression Targets |
|  | IL1B -> NO Expression Targets |
|  | TLR1 -> 2/6 Expression Targets |
|  | IL1B Expression Targets |
|  | TLR4 -> NF-kB/IRF Expression Targets |
|  | IL1A Expression Targets |
|  | TLR9 Expression Targets |
|  | ROS in Triggering Vascular Inflammation |
|  | Elevated Receptors -> Expression Targets in Kidney |
|  | pathway 4 |
|  | PPAR Psoriasis |
|  | prarg negative regulators, ps-positive |
|  | pprarg neg, uknown targets, ps-positive |
|  | prarg neg,ukn expres targets, ps-positive |
|  | PPARG negative regulators |
|  | PPARG negative targets |
|  | pparg expr target\_regulators |
|  | Neighbors of disease exacerbation |
|  | Neighbors of tumor progression |
|  | Neighbors of cancer progression |
|  | Neighbors of inflammatory status |
|  | Neighbors of overall survival |
|  | Neighbors of disease severity |
|  | Neighbors of tumor size |
|  | Neighbors of lesion size |
|  | Neighbors of mortality |
|  | Neighbors of survival rate |
|  | Neighbors of infarct size |
|  | Neighbors of survival time |
|  | Neighbors of functional recovery |
|  | Neighbors of insulin sensitivity |
|  | Neighbors of bacterial load |
|  | Neighbors of cancer survival |
|  | Neighbors of graft survival |
|  | Neighbors of therapeutic efficacy |
|  | Neighbors of clinical stage |
|  | Neighbors of lethality |
|  | Neighbors of tumor regression |
|  | Neighbors of recurrence-free survival |
|  | Neighbors of disease-free survival |
|  | Neighbors of tumor response |
|  | Neighbors of macrophage count |
|  | Neighbors of long-term survival |
|  | Neighbors of treatment outcome |
|  | Neighbors of bone mass |
|  | Neighbors of body weight |
|  | Neighbors of body weight gain |
|  | Neighbors of cardiovascular risk |
|  | Neighbors of microvessel density |
|  | Neighbors of tumor recurrence |
|  | Neighbors of tumor promotion |
|  | Neighbors of neutrophil count |
|  | Neighbors of glucose tolerance |
|  | Neighbors of morbidity |
|  | Neighbors of fibrosis progression |
|  | Neighbors of mortality rate |
|  | Neighbors of fat mass |
|  | Neighbors of exhaustion |
|  | Neighbors of bone mineral density |
|  | Neighbors of adverse outcome |
|  | Neighbors of radioresistance |
|  | Neighbors of lesion area |
|  | Neighbors of cell density |
|  | Neighbors of progression-free survival |
|  | Neighbors of apoptosis rate |
|  | Neighbors of early mortality |
|  | Neighbors of shear stress |
|  | Neighbors of viral load |
|  | Neighbors of blood pressure |
|  | Neighbors of leukocyte count |
|  | Neighbors of tumor grade |
|  | Neighbors of vascular reactivity |
|  | Neighbors of vascular leakage |
|  | Neighbors of body mass index |
|  | Neighbors of vascular density |
|  | Neighbors of muscle mass |
|  | Neighbors of cancer risk |
|  | Neighbors of cancer incidence |
|  | Neighbors of tumor aggressiveness |
|  | Neighbors of systolic blood pressure |
|  | Neighbors of tumor resistance |
|  | Neighbors of blood glucose |
|  | Neighbors of ejection fraction |
|  | Neighbors of median survival |
|  | Neighbors of renal hypertrophy |
|  | Neighbors of fasting plasma glucose |
|  | Neighbors of osteoclast number |
|  | Neighbors of cancer recurrence |
|  | Neighbors of T-cell count |
|  | Neighbors of energy expenditure |
|  | Neighbors of glucose level |
|  | Neighbors of vascular tone |
|  | Neighbors of radiosensitivity |
|  | Neighbors of left ventricular ejection fraction |
|  | Neighbors of breast cancer risk |
|  | Neighbors of proliferation index |
|  | Neighbors of cell permeability |
|  | Neighbors of pregnancy outcome |
|  | Neighbors of functional status |
|  | Neighbors of gastric cancer risk |
|  | Neighbors of neurological outcome |
|  | Neighbors of infarct area |
|  | Neighbors of fertility |
|  | Neighbors of recurrence rate |
|  | Neighbors of pathological response |
|  | Neighbors of lymphocyte count |
|  | Neighbors of monocyte count |
|  | Neighbors of skin thickness |
|  | Neighbors of metabolic response |
|  | Neighbors of insulin level |
|  | Neighbors of comorbidity |
|  | Neighbors of carotid intima-media thickness |
|  | Neighbors of bone volume |
|  | Neighbors of fibroblast phenotype |
|  | Neighbors of fractional shortening |
|  | Neighbors of disease exacerbation |
|  | Neighbors of tumor progression |
|  | Neighbors of cancer progression |
|  | Neighbors of inflammatory status |
|  | Neighbors of overall survival |
|  | Neighbors of disease severity |
|  | Neighbors of tumor size |
|  | Neighbors of lesion size |
|  | Neighbors of mortality |
|  | Neighbors of survival rate |
|  | Neighbors of infarct size |
|  | Neighbors of survival time |
|  | Neighbors of functional recovery |
|  | Neighbors of insulin sensitivity |
|  | Neighbors of bacterial load |
|  | Neighbors of cancer survival |
|  | Neighbors of graft survival |
|  | Neighbors of therapeutic efficacy |
|  | Neighbors of clinical stage |
|  | Neighbors of lethality |
|  | Neighbors of tumor regression |
|  | Neighbors of recurrence-free survival |
|  | Neighbors of disease-free survival |
|  | Neighbors of tumor response |
|  | Neighbors of macrophage count |
|  | Neighbors of long-term survival |
|  | Neighbors of treatment outcome |
|  | Neighbors of bone mass |
|  | Neighbors of body weight |
|  | Neighbors of body weight gain |
|  | Neighbors of cardiovascular risk |
|  | Neighbors of microvessel density |
|  | Neighbors of tumor recurrence |
|  | Neighbors of tumor promotion |
|  | Neighbors of neutrophil count |
|  | Neighbors of glucose tolerance |
|  | Neighbors of morbidity |
|  | Neighbors of fibrosis progression |
|  | Neighbors of mortality rate |
|  | Neighbors of fat mass |
|  | Neighbors of exhaustion |
|  | Neighbors of bone mineral density |
|  | Neighbors of adverse outcome |
|  | Neighbors of radioresistance |
|  | Neighbors of lesion area |
|  | Neighbors of cell density |
|  | Neighbors of progression-free survival |
|  | Neighbors of apoptosis rate |
|  | Neighbors of early mortality |
|  | Neighbors of shear stress |
|  | Neighbors of viral load |
|  | Neighbors of blood pressure |
|  | Neighbors of leukocyte count |
|  | Neighbors of tumor grade |
|  | Neighbors of vascular reactivity |
|  | Neighbors of vascular leakage |
|  | Neighbors of body mass index |
|  | Neighbors of vascular density |
|  | Neighbors of muscle mass |
|  | Neighbors of cancer risk |
|  | Neighbors of cancer incidence |
|  | Neighbors of tumor aggressiveness |
|  | Neighbors of systolic blood pressure |
|  | Neighbors of tumor resistance |
|  | Neighbors of blood glucose |
|  | Neighbors of ejection fraction |
|  | Neighbors of median survival |
|  | Neighbors of renal hypertrophy |
|  | Neighbors of fasting plasma glucose |
|  | Neighbors of osteoclast number |
|  | Neighbors of cancer recurrence |
|  | Neighbors of T-cell count |
|  | Neighbors of energy expenditure |
|  | Neighbors of glucose level |
|  | Neighbors of vascular tone |
|  | Neighbors of radiosensitivity |
|  | Neighbors of left ventricular ejection fraction |
|  | Neighbors of breast cancer risk |
|  | Neighbors of proliferation index |
|  | Neighbors of cell permeability |
|  | Neighbors of pregnancy outcome |
|  | Neighbors of functional status |
|  | Neighbors of gastric cancer risk |
|  | Neighbors of neurological outcome |
|  | Neighbors of infarct area |
|  | Neighbors of fertility |
|  | Neighbors of recurrence rate |
|  | Neighbors of pathological response |
|  | Neighbors of lymphocyte count |
|  | Neighbors of monocyte count |
|  | Neighbors of skin thickness |
|  | Neighbors of metabolic response |
|  | Neighbors of insulin level |
|  | Neighbors of comorbidity |
|  | Neighbors of carotid intima-media thickness |
|  | Neighbors of bone volume |
|  | Neighbors of fibroblast phenotype |
|  | Neighbors of fractional shortening |
|  | Neighbors of cell development |
|  | Neighbors of immune response |
|  | Neighbors of cell population |
|  | Neighbors of T-cell development |
|  | Neighbors of inflammatory response |
|  | Neighbors of wound healing |
|  | Neighbors of angiogenesis |
|  | Neighbors of innate immune response |
|  | Neighbors of cell function |
|  | Neighbors of macrophage activation |
|  | Neighbors of cell formation |
|  | Neighbors of adaptive immune response |
|  | Neighbors of osteoclast development |
|  | Neighbors of epithelial to mesenchymal transition |
|  | Neighbors of immunity |
|  | Neighbors of cell proliferative response |
|  | Neighbors of chemotaxis |
|  | Neighbors of dendritic cell differentiation |
|  | Neighbors of cell survival |
|  | Neighbors of osteoclast differentiation |
|  | Neighbors of cell phenotype |
|  | Neighbors of T-cell proliferation |
|  | Neighbors of tissue remodeling |
|  | Neighbors of cell adhesion |
|  | Neighbors of T-cell activation |
|  | Neighbors of endothelial cell proliferation |
|  | Neighbors of fibrogenesis |
|  | Neighbors of tumor growth |
|  | Neighbors of dendritic cell development |
|  | Neighbors of tissue repair |
|  | Neighbors of cell count |
|  | Neighbors of cell infiltration |
|  | Neighbors of cell interaction |
|  | Neighbors of SMC proliferation |
|  | Neighbors of lumen formation |
|  | Neighbors of endothelial cell migration |
|  | Neighbors of adipogenesis |
|  | Neighbors of lipid storage |
|  | Neighbors of cell damage |
|  | Neighbors of pregnancy |
|  | Neighbors of monocyte differentiation |
|  | Neighbors of aging |
|  | Neighbors of cell motility |
|  | Neighbors of bone resorption |
|  | Neighbors of monocyte migration |
|  | Neighbors of fibroblast proliferation |
|  | Neighbors of adipocyte differentiation |
|  | Neighbors of macrophage migration |
|  | Neighbors of microglial activation |
|  | Neighbors of neutrophil recruitment |
|  | Neighbors of regeneration |
|  | Neighbors of cell migration |
|  | Neighbors of cancer growth |
|  | Neighbors of stem cell proliferation |
|  | Neighbors of smooth muscle cell migration |
|  | Neighbors of hepatic regeneration |
|  | Neighbors of transendothelial migration |
|  | Neighbors of phagocytosis |
|  | Neighbors of immune system activation |
|  | Neighbors of leukocyte migration |
|  | Neighbors of leukocyte recruitment |
|  | Neighbors of osteoclast formation |
|  | Neighbors of monocyte recruitment |
|  | Neighbors of cell invasion |
|  | Neighbors of heart function |
|  | Neighbors of hemopoiesis |
|  | Neighbors of bone remodeling |
|  | Neighbors of ossification |
|  | Neighbors of T-cell function |
|  | Neighbors of immune system function |
|  | Neighbors of lung development |
|  | Neighbors of autophagy |
|  | Neighbors of monocyte adhesion |
|  | Neighbors of transcription activation |
|  | Neighbors of senescence |
|  | Neighbors of T-cell response |
|  | Neighbors of tumor immunity |
|  | Neighbors of hepatocyte proliferation |
|  | Neighbors of cell transdifferentiation |
|  | Neighbors of neuroprotection |
|  | Neighbors of stem cell differentiation |
|  | Neighbors of endothelial cell function |
|  | Neighbors of neuronal death |
|  | Neighbors of cell growth |
|  | Neighbors of ROS generation |
|  | Neighbors of epithelial cell proliferation |
|  | Neighbors of first trimester pregnancy |
|  | Neighbors of cancer cell growth |
|  | Neighbors of sensitization |
|  | Neighbors of hemato-encephalic barrier |
|  | Neighbors of macrophage function |
|  | Neighbors of cellular immune response |
|  | Neighbors of cellular senescence |
|  | Neighbors of colony formation |
|  | Neighbors of macrophage differentiation |
|  | Neighbors of cell differentiation |
|  | Neighbors of keratinocyte proliferation |
|  | Neighbors of stem cell migration |
|  | Neighbors of life span |
|  | Neighbors of cell homeostasis |
|  | Neighbors of psoriasis |
|  | Neighbors of inflammatory disease |
|  | Neighbors of rheumatoid arthritis |
|  | Neighbors of atherosclerosis |
|  | Neighbors of fibrosis |
|  | Neighbors of colitis |
|  | Neighbors of arthritis |
|  | Neighbors of inflammatory bowel disease |
|  | Neighbors of injury |
|  | Neighbors of atherogenesis |
|  | Neighbors of vascular remodeling |
|  | Neighbors of metastasis |
|  | Neighbors of carcinogenesis |
|  | Neighbors of multiple sclerosis |
|  | Neighbors of chronic inflammation |
|  | Neighbors of inflammation |
|  | Neighbors of tumor microenvironment |
|  | Neighbors of pathological angiogenesis |
|  | Neighbors of obesity |
|  | Neighbors of vascular disease |
|  | Neighbors of diabetes mellitus |
|  | Neighbors of asthma |
|  | Neighbors of pneumonia |
|  | Neighbors of hyperplasia |
|  | Neighbors of osteoarthritis |
|  | Neighbors of liver fibrosis |
|  | Neighbors of chronic obstructive pulmonary disease |
|  | Neighbors of myocardial infarction |
|  | Neighbors of leukocyte infiltration |
|  | Neighbors of reperfusion injury |
|  | Neighbors of airway inflammation |
|  | Neighbors of hepatitis |
|  | Neighbors of insulin resistance |
|  | Neighbors of infection |
|  | Neighbors of vascular injury |
|  | Neighbors of renal injury |
|  | Neighbors of preeclampsia |
|  | Neighbors of macrophage infiltration |
|  | Neighbors of liver injury |
|  | Neighbors of neuroinflammation |
|  | Neighbors of sepsis |
|  | Neighbors of death |
|  | Neighbors of type 1 diabetes |
|  | Neighbors of encephalomyelitis |
|  | Neighbors of autoimmune disease |
|  | Neighbors of ischemic stroke |
|  | Neighbors of dermatitis |
|  | Neighbors of neutrophil infiltration |
|  | Neighbors of heart disease |
|  | Neighbors of diabetic nephropathy |
|  | Neighbors of lung injury |
|  | Neighbors of immunopathology |
|  | Neighbors of systemic sclerosis |
|  | Neighbors of hepatocellular carcinoma |
|  | Neighbors of autoimmunity |
|  | Neighbors of acute lung injury |
|  | Neighbors of gastric cancer |
|  | Neighbors of atherosclerotic plaque |
|  | Neighbors of experimental autoimmune encephalomyelitis |
|  | Neighbors of colorectal cancer |
|  | Neighbors of cardiac remodeling |
|  | Neighbors of breast cancer |
|  | Neighbors of ischemia |
|  | Neighbors of systemic lupus erythematosus |
|  | Neighbors of melanoma |
|  | Neighbors of collagen-induced arthritis |
|  | Neighbors of cartilage degeneration |
|  | Neighbors of interstitial fibrosis |
|  | Neighbors of neoplasm invasion |
|  | Neighbors of kidney disease |
|  | Neighbors of heart failure |
|  | Neighbors of lung disease |
|  | Neighbors of pulmonary fibrosis |
|  | Neighbors of cardiovascular disease |
|  | Neighbors of stroke |
|  | Neighbors of cancer |
|  | Neighbors of cerebral ischemia |
|  | Neighbors of type 2 diabetes |
|  | Neighbors of neoplasm |
|  | Neighbors of endometriosis |
|  | Neighbors of neointima |
|  | Neighbors of vasculitis |
|  | Neighbors of atrophy |
|  | Neighbors of diet-induced obesity |
|  | Neighbors of respiratory hypersensitivity |
|  | Neighbors of myocardial fibrosis |
|  | Neighbors of synovitis |
|  | Neighbors of coronary artery disease |
|  | Neighbors of rupture |
|  | Neighbors of endothelial cell dysfunction |
|  | Neighbors of liver disease |
|  | Neighbors of prostate cancer |
|  | Neighbors of hypertrophy |
|  | Neighbors of osteolysis |
|  | Neighbors of ovarian cancer |
|  | Neighbors of pancreatic cancer |
|  | Neighbors of hypertension |
|  | Neighbors of intimal hyperplasia |
|  | Neighbors of allograft rejection |
|  | Neighbors of proteinuria |
|  | Neighbors of epithelial cell |
|  | Neighbors of keratinocyte |
|  | Neighbors of vascular smooth muscle cell |
|  | Neighbors of umbilical vein endothelial cell |
|  | Neighbors of monocyte |
|  | Neighbors of endometrial stromal cell |
|  | Neighbors of alveolar macrophage |
|  | Neighbors of synoviocyte |
|  | Neighbors of macrophage |
|  | Neighbors of adipocyte |
|  | Neighbors of activated monocyte |
|  | Neighbors of fibroblast |
|  | Neighbors of airway epithelial cell |
|  | Neighbors of PBMC |
|  | Neighbors of stromal cell |
|  | Neighbors of airway smooth muscle cell |
|  | Neighbors of osteoblast |
|  | Neighbors of pneumocyte |
|  | Neighbors of endometrium cell |
|  | Neighbors of microglia |
|  | Neighbors of trophoblast |
|  | Neighbors of intestine epithelium cell |
|  | Neighbors of mononuclear cell |
|  | Neighbors of hepatic stellate cell |
|  | Neighbors of hepatocyte |
|  | Neighbors of decidual cell |
|  | Neighbors of kidney mesangial cell |
|  | Neighbors of endothelial cell |
|  | Neighbors of dendritic cell |
|  | Neighbors of Kuppfer cell |
|  | Neighbors of proadipocyte |
|  | Neighbors of kidney tubule cell |
|  | Neighbors of inflammatory cell |
|  | Neighbors of bone marrow macrophage |
|  | Neighbors of peritoneal macrophage |
|  | Neighbors of osteoclast |
|  | Neighbors of neutrophil |
|  | Neighbors of smooth muscle myocyte |
|  | Neighbors of astrocyte |
|  | Neighbors of bone marrow derived dendritic cell |
|  | Neighbors of lung fibroblast |
|  | Neighbors of activated T-cell |
|  | Neighbors of mesothelial cell |
|  | Neighbors of mononuclear phagocyte |
|  | Neighbors of capillary endothelial cell |
|  | Neighbors of immunocompetent cell |
|  | Neighbors of granulocyte |
|  | Neighbors of cardiac myocyte |
|  | Neighbors of cancer-associated fibroblast |
|  | Neighbors of myeloid cell |
|  | Neighbors of mesenchymal stroma cell |
|  | Neighbors of corneal epithelial cell |
|  | Neighbors of cytotrophoblast |
|  | Neighbors of stem cell |
|  | Neighbors of tumor infiltrating macrophage |
|  | Neighbors of sinusoidal cell |
|  | Neighbors of B-cell |
|  | Neighbors of granulosa cell |
|  | Neighbors of leukocyte |
|  | Neighbors of chondrocyte |
|  | Neighbors of insulin-secreting cell |
|  | Neighbors of mesenchymal cell |
|  | Neighbors of T-cell |
|  | Neighbors of M1 macrophage |
|  | Neighbors of myofibroblast |
|  | Neighbors of pancreatic stellate cell |
|  | Neighbors of mast cell |
|  | Neighbors of bone marrow stromal cell |
|  | Neighbors of spleen cell |
|  | Neighbors of brain capillary endothelial cell |
|  | Neighbors of neuroglia |
|  | Neighbors of thyroid epithelial cell |
|  | Neighbors of aortic smooth muscle cell |
|  | Neighbors of hematopoietic cell |
|  | Neighbors of adipose macrophage |
|  | Neighbors of podocyte |
|  | Neighbors of mammary epithelial cell |
|  | Neighbors of conventional dendritic cell |
|  | Neighbors of pericyte |
|  | Neighbors of antigen-presenting cell |
|  | Neighbors of epidermis cell |
|  | Neighbors of bone marrow derived mast cell |
|  | Neighbors of phagocyte |
|  | Neighbors of kidney cell |
|  | Neighbors of syncytiotrophoblast |
|  | Neighbors of lymphocyte |
|  | Neighbors of Langerhans cell |
|  | Neighbors of decidual natural killer cell |
|  | Neighbors of thymic epithelial cell |
|  | Neighbors of brain cell |
|  | Neighbors of M2 macrophage |
|  | Neighbors of peritoneum cell |
|  | Neighbors of lymphoid cell |
|  | Neighbors of retina cell |
|  | Neighbors of blood cell |
|  | Neighbors of skin cell |
|  | Neighbors of luteal cell |
|  | Neighbors of peripheral lymphocyte |
|  | Neighbors of activated B-cell |
|  | Neighbors of lymph node cell |
|  | Neighbors of lung |
|  | Neighbors of liver |
|  | Neighbors of kidney |
|  | Neighbors of skin |
|  | Neighbors of intestine |
|  | Neighbors of lymph node |
|  | Neighbors of colon |
|  | Neighbors of airway |
|  | Neighbors of blood vessel |
|  | Neighbors of heart |
|  | Neighbors of aorta |
|  | Neighbors of gastrointestinal tract |
|  | Neighbors of microvessel |
|  | Neighbors of bone |
|  | Neighbors of placenta |
|  | Neighbors of carotid artery |
|  | Neighbors of synovial membrane |
|  | Neighbors of uterus |
|  | Neighbors of esophagus |
|  | Neighbors of periodontium |
|  | Neighbors of retina |
|  | Neighbors of brain |
|  | Neighbors of ovary |
|  | Neighbors of breast |
|  | Neighbors of blood vessel wall |
|  | Neighbors of stomach |
|  | Neighbors of islets of Langerhans |
|  | Neighbors of joint |
|  | Neighbors of artery |
|  | Neighbors of cardiovascular system |
|  | Neighbors of pancreas |
|  | Neighbors of prostate |
|  | Neighbors of central nervous system |
|  | Neighbors of kidney glomerulus |
|  | Neighbors of lymphatic system |
|  | Neighbors of colorectal region |
|  | Neighbors of decidua |
|  | Neighbors of amnion |
|  | Neighbors of immune system |
|  | Neighbors of spleen |
|  | Neighbors of peritoneum |
|  | Neighbors of allantois |
|  | Neighbors of alveolus |
|  | Neighbors of coronary artery |
|  | Neighbors of left ventricle |
|  | Neighbors of uterine cervix |
|  | Neighbors of omentum |
|  | Neighbors of vein |
|  | Neighbors of villus |
|  | Neighbors of bronchus |
|  | Neighbors of glands |
|  | Neighbors of eye |
|  | Neighbors of cornea |
|  | Neighbors of hippocampus |
|  | Neighbors of intervertebral disc |
|  | Neighbors of lymph node of mesentery |
|  | Neighbors of mammary gland |
|  | Neighbors of vagina |
|  | Neighbors of ankle joint |
|  | Neighbors of thymus gland |
|  | Neighbors of cerebral cortex |
|  | Neighbors of brain blood vessel |
|  | Neighbors of myelin sheath |
|  | Neighbors of bladder |
|  | Neighbors of draining lymph node |
|  | Neighbors of nervous system |
|  | Neighbors of retina blood vessel |
|  | Neighbors of mesentery |
|  | Neighbors of nasopharynx |
|  | Neighbors of salivary gland |
|  | Neighbors of trachea |
|  | Neighbors of corpus luteum of ovary |
|  | Neighbors of sympathetic nervous system |
|  | Neighbors of nerve |
|  | Neighbors of ileum |
|  | Neighbors of endocrine system |
|  | Neighbors of lower extremity |
|  | Neighbors of small intestine |
|  | Neighbors of aggregated lymphoid follicle of small intestine |
|  | Neighbors of globus pallidus |
|  | Neighbors of amniotic cavity |
|  | Neighbors of lumbar spine |
|  | Neighbors of knee joint |
|  | Neighbors of pleura |
|  | Neighbors of left atrium |
|  | Neighbors of Fallopian tube |
|  | Neighbors of hypothalamus |
|  | Neighbors of hair follicle |
|  | Neighbors of artery wall |
|  | Neighbors of NF-kB family |
|  | Neighbors of oxidized LDL |
|  | Neighbors of JNK |
|  | Neighbors of IL1 family |
|  | Neighbors of PPAR |
|  | Neighbors of Jun/Fos |
|  | Neighbors of NFKBI |
|  | Neighbors of PKC |
|  | Neighbors of PI3K |
|  | Neighbors of STAT family |
|  | Neighbors of mitogen-activated protein kinase |
|  | Neighbors of IFNAR ligand |
|  | Neighbors of transforming growth factor |
|  | Neighbors of TLR |
|  | Neighbors of cytokine |
|  | Neighbors of interferon |
|  | Neighbors of interleukin |
|  | Neighbors of JAK |
|  | Neighbors of MEK1/2 |
|  | Neighbors of PDGF |
|  | Neighbors of Ras GTPase |
|  | Neighbors of ERK1/2 |
|  | Neighbors of growth factor |
|  | Neighbors of PKA |
|  | Neighbors of ovalbumin |
|  | Neighbors of LDL |
|  | Neighbors of NF-AT family |
|  | Neighbors of beta adrenoceptor |
|  | Neighbors of chorionic gonadotropin |
|  | Neighbors of IKBK |
|  | Neighbors of adipokine |
|  | Neighbors of protein tyrosine kinase |
|  | Neighbors of NAD(P)H oxidase |
|  | Neighbors of IgG |
|  | Neighbors of AMPK |
|  | Neighbors of LXR |
|  | Neighbors of HDL |
|  | Neighbors of p38 |
|  | Neighbors of inflammatory cytokine |
|  | Neighbors of IL23 |
|  | Neighbors of estrogen receptor |
|  | Neighbors of T-cell receptor |
|  | Neighbors of histone deacetylase |
|  | Neighbors of PDGF-BB |
|  | Neighbors of IL1R |
|  | Neighbors of GPCR |
|  | Neighbors of IL12 |
|  | Neighbors of Notch |
|  | Neighbors of prostaglandin-endoperoxide synthase |
|  | Neighbors of heat shock protein 90 |
|  | Neighbors of lectin |
|  | Neighbors of fibrinogen |
|  | Neighbors of aldehyde reductase |
|  | Neighbors of mitogen-activated protein kinase kinase |
|  | Neighbors of interleukin-35 |
|  | Neighbors of TSH |
|  | Neighbors of Ang II receptor |
|  | Neighbors of endothelin |
|  | Neighbors of superoxide dismutase |
|  | Neighbors of proteasome endopeptidase complex |
|  | Neighbors of class nuclear receptor with C4 zinc fingers |
|  | Neighbors of non-coding RNA |
|  | Neighbors of histone H3 |
|  | Neighbors of STAT5 |
|  | Neighbors of alpha2 adrenoceptor |
|  | Neighbors of vitronectin receptor (integrin) |
|  | Neighbors of PP2A |
|  | Neighbors of retinoid-X receptor subfamily |
|  | Neighbors of SAPK |
|  | Neighbors of WNT |
|  | Neighbors of gonadotropins |
|  | Neighbors of immunoglobulin |
|  | Neighbors of PP2B |
|  | Neighbors of phospholipase |
|  | Neighbors of endostatins |
|  | Neighbors of CD3 complex |
|  | Neighbors of relaxin |
|  | Neighbors of TLR1/2 heterodimer |
|  | Neighbors of FSH |
|  | Neighbors of TORC1 |
|  | Neighbors of angiotensin receptor |
|  | Neighbors of RA receptor |
|  | Neighbors of histone |
|  | Neighbors of Rho kinase |
|  | Neighbors of PDGF receptor |
|  | Neighbors of neuropeptides |
|  | Neighbors of pancreatic elastase |
|  | Neighbors of histone acetyltransferase |
|  | Neighbors of adrenoceptor |
|  | Neighbors of phosphodiesterase |
|  | Neighbors of caspase |
|  | Neighbors of phosphoinositide phospholipase C |
|  | Neighbors of deacetylase |
|  | Neighbors of TLR4/Myd88 |
|  | Neighbors of TNF-alpha receptor |
|  | comon ps\_pos, pprarg\_neg targets |
|  | Model of PPARG signaling in psoriasis |
|  | PPARG negative regulators and targets |
|  | Model of PPARG related pathways in psoriasis (short version) |
|  | PPARs and anxiety common targets |
|  | Amygdaloid expressed genes |
|  | PPARs and neuroinflamation |
|  | sars-cov no binding |
|  | New Pathway (4) |
|  | dsRNA induces innate immunity and INFs in alveolar cells |
|  | Dendritic Cell Activation in COVID19 |
|  | Overview. Diseases of the skin (chapter 11) |
|  | Overview. Diseases of the digestive system (chapter 10) |
|  | Overview. Diseases of the nervous system (chapter 5) |
|  | Overview. Diseases of the eye (chapter 6) |
|  | Overview. Diseases of the respiratory (chapter 9) |
|  | Overview. Diseases of the genitourinary system (chapter 13) |
|  | Overview. Endocrine and metabolic diseases (chapter 4) |
|  | Overview. Infectious and parasitic diseases (chapter 2) |
|  | 1\_Inflammation in keratinocytes in the pathology of acne |
|  | 2\_Acute phase initiation in atopic dermatitis |
|  | 1\_Differentiation of psoriatic T cells |
|  | 1\_TNF and IL1B provoke ECM degradation in osteoarthritis |
|  | 1\_Osteoclast-mediated bone resorption in postmenopausal women |
|  | 2\_Chondrocyte death in osteoarthritis |
|  | 1\_The role of Toll-like receptors in pyelonephritis |
|  | 2\_The role of endothelial cells in pyelonephritis |
|  | 3\_The role of interstitial fibroblasts in pyelonephritis |
|  | 2\_Podocyte dysfunction in glomerulonephritis |
|  | 1\_Insufficient activation of immune response in the middle ear epithelium cells in otitis media |
|  | 2\_Pathogens stimulate mucins expression in the middle ear |
|  | 2\_Cardiomyocyte hypertrophy |
|  | 1\_Cigarette smoke causes dysfunction and production of inflammatory mediators in alveolar epithelial cells |
|  | 2\_Cigarette smoke and inflammation cause alveolar epithelial cell death in COPD |
|  | 3\_Role of alveolar macrophages in pulmonary tissue destruction in COPD |
|  | 1\_Th2 cell response in asthma |
|  | 3\_2\_Regulation of airway surface liquid (ASL) thickness |
|  | 3\_1\_Mucus accumulation in asthma |
|  | 4\_Mucin hyperproduction in goblet and mucous cells in COPD |
|  | 1\_Defects in response to pathogens in the gut promotes inflammation in Crohn's disease |
|  | 2\_Intestine epithelial cells drive immunological responses in ulcerative colitis |
|  | 1\_Intestine epithelial cell dysfunction in ulcerative colitis |
|  | 4\_1\_Polymorphisms associated with inflammatory bowel diseases |
|  | 2\_Lipotoxicity in NAFLD |
|  | 6\_p12I Viral protein promotes T-cell activation in HTLV-1 infection |
|  | 5\_Human papilloma virus E6 and E7 proteins promote epithelial cell proliferation |
|  | 2\_HIV - induced depletion of CD4+ T-cells |
|  | 3\_Impairment of iron release from splenic macrophages |
|  | 5\_The rationale for the treatment of anemia of chronic disease with erythropoietin |
|  | 4\_Inflammatory mediators inhibit GATA signaling in erythropoiesis |
|  | 4\_2\_Insulin resistance: inflammation-related insulin resistance |
|  | 1\_Antigen-presenting cells promote islet dysfunction and immune system activation in T1D |
|  | 2\_Neuron and Schwann cell death |
|  | Figure 4\_Diabetes mellitus Type 1: conventional dendritic cell |
|  | drug-target pairs binding |
|  | drug-target pairs direct regulation |
|  | drug-target moltransport |
|  | drug-target molsynthesis |
|  | New Combined Pathway |
|  | Differentiation of psoriatic T cells |

---

|  |  |
| --- | --- |
| Group | Genes with Mutations Associated with Atherosclerosis |
|  | Genes with Mutations Associated with Psoriasis |
|  | Genes Associated with Systemic Lupus Erythematosus |
|  | Genes with Mutations Associated with Vitiligo |
|  | Proteins Involved in Graves Ophthalmopathy |
|  | Genes with Mutations Associated with Obesity |
|  | Genes Associated with Ulcerative Colitis |
|  | Genes with Mutations Associated with Asthma |
|  | Genes with Mutations Associated with Psoriatic Arthritis |
|  | Genes with Mutations Associated with Systemic Scleroderma |
|  | Genes with Polymorphisms Associated with Migraine |
|  | Genes with Mutations Associated with Multiple Sclerosis |
|  | Genes with Mutations Associated with Endometriosis |
|  | other partners for SARS |

---

|  |  |
| --- | --- |
| MedScan ID | 7124 |

---

|  |  |
| --- | --- |
| LocusLink ID | 7124 |
|  | 21926 |
|  | 24835 |
|  | 257601 |
|  | 257551 |

---

|  |  |
| --- | --- |
| Alias | TNF alfa |
|  | DADB-70P7.1 |
|  | TNF superfamily, member 2 |
|  | Tnlg1f |
|  | TNF, macrophage-derived |
|  | OTTHUMP00000037669 |
|  | tumor necrotizing factor alpha |
|  | pre-tumor necrosis factor |
|  | MGC124630 |
|  | pro-tumor necrosis factor |
|  | TNFA\_HUMAN |
|  | TNFA |
|  | TNF-a |
|  | LOC257551 |
|  | Tnfsf1a |
|  | tumor necrosis alpha |
|  | tumor necrosis factor alfa |
|  | tumor necrosis factor ligand superfamily member 2 |
|  | TNFSF2 |
|  | TNF-alpha |
|  | proTNF-alpha |
|  | tumor necrosis factor II |
|  | MGC151434 |
|  | tumor necrosis factor 2 |
|  | tumor necrosis factor |
|  | TNF |
|  | OTTHUMP00000029281 |
|  | tumor necrosis factor superfamily member 2 |
|  | tumor necrosis factor alpha |
|  | pro-TNF-alpha |
|  | RATTNF |
|  | protumour necrosis factor |
|  | cachectin |
|  | TNF superfamily, member II |
|  | TNF, monocyte-derived |
|  | protumor necrosis factor |
|  | TNF precursor |
|  | cachectin-tumor necrosis factor propeptide |
|  | tumor necrosis factor ligand 1f |
|  | APC1 gene |
|  | LOC257601 |
|  | DIF |
|  | APC1 protein |
|  | tumor necrosis factor-alpha |
|  | TNFalpha |
|  | tumor necrosis factor (TNF superfamily, member 2) |
|  | tumour necrosis factor 2 |
|  | tumour necrosis factor II |
|  | tumour necrosis factor ligand superfamily member 2 |
|  | tumour necrosis factor superfamily member 2 |
|  | cachectin-tumour necrosis factor propeptide |
|  | tumour necrosis factor |
|  | tumour necrosis factor alpha |
|  | tumour necrotizing factor alpha |
|  | pre-tumour necrosis factor |
|  | tumour necrosis factor alfa |
|  | tumour necrosis factor (TNF superfamily, member 2) |
|  | TNF alpha |
|  | tumor necrosis factor, alpha (cachectin) |
|  | tumor necrosis factor, alpha (cachetin) |
|  | tumour necrosis factor, alpha (cachectin) |
|  | tumour necrosis factor, alpha (cachetin) |
|  | tumor necrosis factor superfamily, member 2 |
|  | tumor necrosis factor superfamily, member 2 |
|  | tumor necrosis factor superfamily member 2 |
|  | TNFSF2s |
|  | tumor necrosis factor, alpha |
|  | tnf superfamily |
|  | DASS-280D8.2 |
|  | Tumor necrosis factor alpha (cachetin) |
|  | similar to tumor necrosis factor (tnf superfamily |
|  | similar to tumor necrosis factor (tnf superfamily, member 2) |
|  | alpha (cachetin) |
|  | tumor necrosis factor superfamily member II |

---

|  |  |
| --- | --- |
| GO ID | 0005125 |
|  | 0002020 |
|  | 0044212 |
|  | 0005164 |
|  | 0007249 |
|  | 0007254 |
|  | 0000165 |
|  | 0000187 |
|  | 0000185 |
|  | 0006919 |
|  | 0048143 |
|  | 0071230 |
|  | 0071316 |
|  | 0071407 |
|  | 0002439 |
|  | 0050890 |
|  | 0030866 |
|  | 0019221 |
|  | 0071550 |
|  | 0050830 |
|  | 0048566 |
|  | 0072577 |
|  | 0060664 |
|  | 0030198 |
|  | 0097191 |
|  | 0008625 |
|  | 0006006 |
|  | 0006959 |
|  | 0006954 |
|  | 0008630 |
|  | 0050901 |
|  | 0031663 |
|  | 0001774 |
|  | 0097527 |
|  | 0010693 |
|  | 1900222 |
|  | 1903347 |
|  | 0061048 |
|  | 0002740 |
|  | 0001937 |
|  | 2001240 |
|  | 0045599 |
|  | 0010629 |
|  | 0046325 |
|  | 0044130 |
|  | 0032715 |
|  | 0050995 |
|  | 0010888 |
|  | 0045930 |
|  | 0045662 |
|  | 0035509 |
|  | 0050768 |
|  | 0045668 |
|  | 0043242 |
|  | 0000122 |
|  | 0045892 |
|  | 0045071 |
|  | 0030316 |
|  | 0051091 |
|  | 0070374 |
|  | 0043123 |
|  | 0046427 |
|  | 0043507 |
|  | 0043406 |
|  | 0051092 |
|  | 1901224 |
|  | 1902004 |
|  | 0043065 |
|  | 2000334 |
|  | 0060559 |
|  | 0070886 |
|  | 0045785 |
|  | 2000304 |
|  | 2000343 |
|  | 0045080 |
|  | 0032722 |
|  | 0090197 |
|  | 0002876 |
|  | 0043280 |
|  | 0001819 |
|  | 0050715 |
|  | 0031622 |
|  | 0050754 |
|  | 0010628 |
|  | 0060252 |
|  | 0051798 |
|  | 0034116 |
|  | 0002925 |
|  | 0050729 |
|  | 0032729 |
|  | 0045410 |
|  | 0032755 |
|  | 2000778 |
|  | 0045416 |
|  | 0032757 |
|  | 1904999 |
|  | 1904996 |
|  | 0051044 |
|  | 0071677 |
|  | 0150078 |
|  | 0043525 |
|  | 0045429 |
|  | 0051000 |
|  | 0051173 |
|  | 0045672 |
|  | 0033138 |
|  | 0050766 |
|  | 0014068 |
|  | 0071803 |
|  | 0043068 |
|  | 0045732 |
|  | 0031334 |
|  | 0043243 |
|  | 0051897 |
|  | 0045860 |
|  | 2000010 |
|  | 1903078 |
|  | 0001934 |
|  | 0051222 |
|  | 0048661 |
|  | 1901671 |
|  | 0050806 |
|  | 0045944 |
|  | 0045893 |
|  | 0045994 |
|  | 0042531 |
|  | 1904707 |
|  | 0060557 |
|  | 0043491 |
|  | 0072659 |
|  | 0032800 |
|  | 0043122 |
|  | 0060693 |
|  | 2000351 |
|  | 1903140 |
|  | 0045598 |
|  | 0051023 |
|  | 0050796 |
|  | 0050807 |
|  | 0051966 |
|  | 0006357 |
|  | 0010803 |
|  | 0051384 |
|  | 0009651 |
|  | 0009615 |
|  | 0030730 |
|  | 0033209 |
|  | 0009986 |
|  | 0009897 |
|  | 0005576 |
|  | 0005615 |
|  | 0005887 |
|  | 0045121 |
|  | 0001891 |
|  | 0005886 |
|  | 0055037 |
|  | 0009887 |
|  | 0006915 |
|  | 0097190 |
|  | 0019722 |
|  | 0001775 |
|  | 0045123 |
|  | 0071222 |
|  | 0006952 |
|  | 0042742 |
|  | 0050966 |
|  | 0006955 |
|  | 0050900 |
|  | 0007275 |
|  | 0002037 |
|  | 0008285 |
|  | 0031642 |
|  | 0046330 |
|  | 0045760 |
|  | 0008284 |
|  | 0032741 |
|  | 0045840 |
|  | 0042127 |
|  | 0050727 |
|  | 0045670 |
|  | 0001932 |
|  | 0050708 |
|  | 2000377 |
|  | 0032496 |
|  | 0010033 |
|  | 0007165 |
|  | 0034138 |
|  | 0042311 |
|  | 0005737 |
|  | 0016021 |
|  | 0016020 |
|  | 0043025 |
|  | 0030141 |
|  | 0002526 |
|  | 1904646 |
|  | 0071300 |
|  | 0007623 |
|  | 2000353 |
|  | 2000866 |
|  | 0010469 |
|  | 0014823 |
|  | 0042493 |
|  | 1990268 |
|  | 0001666 |
|  | 0009612 |
|  | 0009314 |
|  | 0003009 |
|  | 0005622 |
|  | 0000060 |
|  | 0008283 |
|  | 0090002 |
|  | 0042346 |
|  | 0051533 |
|  | 0006927 |
|  | 0070265 |
|  | 0010940 |
|  | 0008652 |
|  | 0006917 |
|  | 0060555 |
|  | 0043066 |
|  | 0006916 |
|  | 0008624 |
|  | 0010843 |
|  | 0016563 |
|  | 0016564 |
|  | 0032582 |
|  | 0043193 |
|  | 0010552 |
|  | 0042802 |
|  | 0016481 |
|  | 0045941 |
|  | 0005515 |
|  | 0007159 |
|  | 0005625 |
|  | 0009611 |
|  | 0006355 |
|  | 0006968 |
|  | 0004871 |
|  | 0050875 |
|  | 0007267 |

---

|  |  |
| --- | --- |
| KEGG ID | hsa:7124 |
|  | mmu:21926 |
|  | rno:24835 |

---

|  |  |
| --- | --- |
| MedLine Reference | 10331026 |
|  | 11477479 |
|  | 11562369 |
|  | 11668049 |
|  | 11686853 |
|  | 11687663 |
|  | 11751152 |
|  | 11756405 |
|  | 11770045 |
|  | 11784800 |
|  | 11793965 |
|  | 11799072 |
|  | 11805112 |
|  | 11820455 |
|  | 11834488 |
|  | 11837786 |
|  | 11875120 |
|  | 11910117 |
|  | 11954829 |
|  | 11961311 |
|  | 11973430 |
|  | 12046086 |
|  | 12052444 |
|  | 12057008 |
|  | 12057931 |
|  | 12072413 |
|  | 12077131 |
|  | 12081562 |
|  | 12096921 |
|  | 12097404 |
|  | 12097495 |
|  | 12118012 |
|  | 12122042 |
|  | 12126639 |
|  | 12130632 |
|  | 12132588 |
|  | 12161343 |
|  | 12161450 |
|  | 12167759 |
|  | 12193225 |
|  | 12193577 |
|  | 12353928 |
|  | 12376316 |
|  | 12388149 |
|  | 12388406 |
|  | 12388422 |
|  | 12388423 |
|  | 12388473 |
|  | 12392281 |
|  | 12393899 |
|  | 12438087 |
|  | 12445823 |
|  | 12477932 |
|  | 12478619 |
|  | 12488366 |
|  | 12529282 |
|  | 12600263 |
|  | 12606464 |
|  | 12611391 |
|  | 12622907 |
|  | 12626358 |
|  | 12631113 |
|  | 12644145 |
|  | 12675973 |
|  | 12684435 |
|  | 12700162 |
|  | 12713870 |
|  | 12775721 |
|  | 12782627 |
|  | 12785009 |
|  | 12849708 |
|  | 12876290 |
|  | 12878376 |
|  | 12887130 |
|  | 12960020 |
|  | 12962917 |
|  | 12967639 |
|  | 12969138 |
|  | 14514475 |
|  | 14602083 |
|  | 14606087 |
|  | 14614903 |
|  | 14615050 |
|  | 14623371 |
|  | 14625205 |
|  | 14625460 |
|  | 14651966 |
|  | 14654230 |
|  | 14703611 |
|  | 14709837 |
|  | 14715528 |
|  | 14718719 |
|  | 14752292 |
|  | 14764603 |
|  | 14993492 |
|  | 14999074 |
|  | 15025948 |
|  | 15039305 |
|  | 15060019 |
|  | 15063785 |
|  | 15111313 |
|  | 15149857 |
|  | 15164724 |
|  | 15170075 |
|  | 15210453 |
|  | 15297437 |
|  | 15322069 |
|  | 15381257 |
|  | 15464762 |
|  | 15467353 |
|  | 15476580 |
|  | 15489642 |
|  | 15509749 |
|  | 15538942 |
|  | 15602748 |
|  | 15629429 |
|  | 15652405 |
|  | 15663790 |
|  | 15665035 |
|  | 15707973 |
|  | 15713255 |
|  | 15737463 |
|  | 15765524 |
|  | 15781751 |
|  | 15798949 |
|  | 15818044 |
|  | 15901845 |
|  | 15902099 |
|  | 15920760 |
|  | 15927374 |
|  | 15935092 |
|  | 15935952 |
|  | 15941855 |
|  | 15942680 |
|  | 15944262 |
|  | 15946935 |
|  | 15986200 |
|  | 16002775 |
|  | 16019429 |
|  | 16044092 |
|  | 16123171 |
|  | 16141456 |
|  | 16144982 |
|  | 16173529 |
|  | 16189509 |
|  | 16269520 |
|  | 1627266 |
|  | 16284227 |
|  | 16307187 |
|  | 16319143 |
|  | 16322490 |
|  | 16322532 |
|  | 16328103 |
|  | 16336586 |
|  | 16341933 |
|  | 16354757 |
|  | 16410364 |
|  | 16415373 |
|  | 16431135 |
|  | 16461369 |
|  | 16629649 |
|  | 16675114 |
|  | 16680015 |
|  | 16698033 |
|  | 16730193 |
|  | 16762003 |
|  | 16813528 |
|  | 16840655 |
|  | 16864744 |
|  | 16874877 |
|  | 16882655 |
|  | 16886721 |
|  | 16923814 |
|  | 16926404 |
|  | 16934751 |
|  | 16949134 |
|  | 17009398 |
|  | 17046730 |
|  | 17048691 |
|  | 17053043 |
|  | 17070605 |
|  | 17109621 |
|  | 17151266 |
|  | 1889806 |
|  | 70317 |
|  | 8224868 |
|  | 9590569 |
|  | 9779826 |
|  | 10064079 |
|  | 10077625 |
|  | 10080539 |
|  | 10082135 |
|  | 10092079 |
|  | 10190896 |
|  | 10204494 |
|  | 10211990 |
|  | 10212002 |
|  | 10224059 |
|  | 10226804 |
|  | 10329594 |
|  | 10339421 |
|  | 10349636 |
|  | 10395330 |
|  | 10415034 |
|  | 10415063 |
|  | 10416582 |
|  | 10417271 |
|  | 10444590 |
|  | 10452966 |
|  | 10452974 |
|  | 10456884 |
|  | 10458761 |
|  | 10469308 |
|  | 10473584 |
|  | 10476056 |
|  | 10477548 |
|  | 10493498 |
|  | 10496522 |
|  | 10514016 |
|  | 10517960 |
|  | 10521784 |
|  | 10528208 |
|  | 10540319 |
|  | 10540321 |
|  | 10562322 |
|  | 10562323 |
|  | 10585765 |
|  | 10586051 |
|  | 10608881 |
|  | 10614649 |
|  | 10615069 |
|  | 10620607 |
|  | 10637268 |
|  | 10639149 |
|  | 10657611 |
|  | 10689296 |
|  | 10692238 |
|  | 10698075 |
|  | 10702415 |
|  | 10704240 |
|  | 10708760 |
|  | 10783129 |
|  | 10783158 |
|  | 10801288 |
|  | 10805971 |
|  | 10878503 |
|  | 10899905 |
|  | 10899912 |
|  | 10900348 |
|  | 10917533 |
|  | 10919279 |
|  | 10919290 |
|  | 10926756 |
|  | 10940885 |
|  | 10940893 |
|  | 10952728 |
|  | 11002334 |
|  | 11042159 |
|  | 11053690 |
|  | 11063830 |
|  | 11067918 |
|  | 11076861 |
|  | 11085746 |
|  | 11123309 |
|  | 11133507 |
|  | 11159184 |
|  | 11163183 |
|  | 11181695 |
|  | 11217851 |
|  | 11222376 |
|  | 11238176 |
|  | 11238648 |
|  | 11239407 |
|  | 11241200 |
|  | 11254358 |
|  | 11275690 |
|  | 11292619 |
|  | 11293812 |
|  | 11301049 |
|  | 11354245 |
|  | 11413159 |
|  | 11433391 |
|  | 11447117 |
|  | 11448951 |
|  | 11455120 |
|  | 11457927 |
|  | 11477542 |
|  | 11477543 |
|  | 11489168 |
|  | 11489971 |
|  | 11500099 |
|  | 11502710 |
|  | 11536150 |
|  | 11564180 |
|  | 11564813 |
|  | 11585627 |
|  | 11588035 |
|  | 11600888 |
|  | 11672536 |
|  | 11673538 |
|  | 11673544 |
|  | 11685224 |
|  | 11707574 |
|  | 11714837 |
|  | 11728336 |
|  | 11734555 |
|  | 11739517 |
|  | 11739537 |
|  | 11751409 |
|  | 11753206 |
|  | 11774542 |
|  | 11781294 |
|  | 11781360 |
|  | 11792832 |
|  | 11792852 |
|  | 11799106 |
|  | 11801629 |
|  | 11809756 |
|  | 11821235 |
|  | 11826115 |
|  | 11826759 |
|  | 11830590 |
|  | 11839803 |
|  | 11847111 |
|  | 11847479 |
|  | 11856733 |
|  | 11865195 |
|  | 11880299 |
|  | 11880364 |
|  | 11882017 |
|  | 11884396 |
|  | 11884480 |
|  | 11897709 |
|  | 11907097 |
|  | 11908709 |
|  | 11917000 |
|  | 11923051 |
|  | 11923482 |
|  | 11923871 |
|  | 11936774 |
|  | 11955948 |
|  | 11959104 |
|  | 11962515 |
|  | 11971010 |
|  | 11986277 |
|  | 11994487 |
|  | 11997234 |
|  | 12011009 |
|  | 12021316 |
|  | 12031976 |
|  | 12031982 |
|  | 12061770 |
|  | 12067206 |
|  | 12068289 |
|  | 12069182 |
|  | 12082611 |
|  | 12083761 |
|  | 12093877 |
|  | 12112371 |
|  | 12115607 |
|  | 12115620 |
|  | 12121864 |
|  | 12126646 |
|  | 12127799 |
|  | 12140188 |
|  | 12142564 |
|  | 12160952 |
|  | 12162877 |
|  | 12163535 |
|  | 12176089 |
|  | 12183573 |
|  | 12184911 |
|  | 12185082 |
|  | 12193562 |
|  | 12196549 |
|  | 12205053 |
|  | 12220546 |
|  | 12221281 |
|  | 12225379 |
|  | 12235115 |
|  | 12270697 |
|  | 12297110 |
|  | 12356572 |
|  | 12360479 |
|  | 12365717 |
|  | 12370360 |
|  | 12370383 |
|  | 12376397 |
|  | 12377103 |
|  | 12388222 |
|  | 12388730 |
|  | 12390309 |
|  | 12391106 |
|  | 12392966 |
|  | 12393292 |
|  | 12393601 |
|  | 12393629 |
|  | 12393670 |
|  | 12393677 |
|  | 12431371 |
|  | 12438325 |
|  | 12438335 |
|  | 12443720 |
|  | 12444124 |
|  | 12446684 |
|  | 12446776 |
|  | 12446781 |
|  | 12447768 |
|  | 12466150 |
|  | 12466851 |
|  | 12466897 |
|  | 12471141 |
|  | 12473067 |
|  | 12483539 |
|  | 12486099 |
|  | 12490655 |
|  | 12496444 |
|  | 12505667 |
|  | 12508119 |
|  | 12519301 |
|  | 12522038 |
|  | 12557750 |
|  | 12560241 |
|  | 12566451 |
|  | 12571077 |
|  | 12584730 |
|  | 12594062 |
|  | 12604029 |
|  | 12610112 |
|  | 12610687 |
|  | 12616536 |
|  | 12619928 |
|  | 12628924 |
|  | 12637573 |
|  | 12651603 |
|  | 12654261 |
|  | 12654642 |
|  | 12654859 |
|  | 12684509 |
|  | 12695331 |
|  | 12697151 |
|  | 12709443 |
|  | 12713579 |
|  | 12727921 |
|  | 12730063 |
|  | 12730147 |
|  | 12734353 |
|  | 12759453 |
|  | 12761567 |
|  | 12773301 |
|  | 12773307 |
|  | 12773432 |
|  | 12787557 |
|  | 12794148 |
|  | 12797522 |
|  | 12801416 |
|  | 12807894 |
|  | 12816730 |
|  | 12817006 |
|  | 12820962 |
|  | 12838504 |
|  | 12842760 |
|  | 12850812 |
|  | 12860530 |
|  | 12867038 |
|  | 12874189 |
|  | 12881420 |
|  | 12882833 |
|  | 12884305 |
|  | 12885938 |
|  | 12907458 |
|  | 12912811 |
|  | 12918124 |
|  | 12929194 |
|  | 12933825 |
|  | 12933876 |
|  | 12933883 |
|  | 12937155 |
|  | 12939259 |
|  | 12941478 |
|  | 12943369 |
|  | 12958312 |
|  | 12960267 |
|  | 12960337 |
|  | 12964046 |
|  | 12968667 |
|  | 12972399 |
|  | 1318227 |
|  | 1351875 |
|  | 13678668 |
|  | 13679037 |
|  | 1371890 |
|  | 1379563 |
|  | 1387105 |
|  | 1390889 |
|  | 1420994 |
|  | 14505032 |
|  | 14511565 |
|  | 14524421 |
|  | 14550262 |
|  | 14558090 |
|  | 14560009 |
|  | 14563845 |
|  | 14578857 |
|  | 14581001 |
|  | 14583241 |
|  | 14585994 |
|  | 14586011 |
|  | 14587096 |
|  | 14593216 |
|  | 14595438 |
|  | 14607912 |
|  | 14617571 |
|  | 14617760 |
|  | 14638845 |
|  | 14638848 |
|  | 14647895 |
|  | 14654378 |
|  | 14654461 |
|  | 14662843 |
|  | 14679059 |
|  | 14688095 |
|  | 14691481 |
|  | 14695318 |
|  | 14706453 |
|  | 14707051 |
|  | 14730625 |
|  | 14733929 |
|  | 14734753 |
|  | 14742542 |
|  | 14743437 |
|  | 14744933 |
|  | 14745016 |
|  | 14749357 |
|  | 14753413 |
|  | 14755337 |
|  | 14764739 |
|  | 14767994 |
|  | 14966082 |
|  | 14969390 |
|  | 14981113 |
|  | 14985352 |
|  | 14988231 |
|  | 14999072 |
|  | 14999690 |
|  | 15009470 |
|  | 1502148 |
|  | 15034066 |
|  | 1503608 |
|  | 15069018 |
|  | 15071180 |
|  | 15075251 |
|  | 15081311 |
|  | 15086382 |
|  | 15100264 |
|  | 15100302 |
|  | 15104249 |
|  | 15126416 |
|  | 15131799 |
|  | 15138266 |
|  | 15140182 |
|  | 15153508 |
|  | 15153522 |
|  | 15155767 |
|  | 15161633 |
|  | 15162501 |
|  | 15162831 |
|  | 15169905 |
|  | 15170228 |
|  | 15175328 |
|  | 15181007 |
|  | 15199050 |
|  | 15199130 |
|  | 15210762 |
|  | 15214027 |
|  | 15240656 |
|  | 15240744 |
|  | 15242735 |
|  | 15245428 |
|  | 15259010 |
|  | 15265903 |
|  | 15270844 |
|  | 15273742 |
|  | 15288121 |
|  | 15289505 |
|  | 15294953 |
|  | 15304360 |
|  | 15311275 |
|  | 15314695 |
|  | 15322203 |
|  | 15322543 |
|  | 15331419 |
|  | 15334086 |
|  | 1533632 |
|  | 15337758 |
|  | 15345516 |
|  | 15347683 |
|  | 15349890 |
|  | 15351034 |
|  | 15358232 |
|  | 15361580 |
|  | 15365619 |
|  | 15366373 |
|  | 15377396 |
|  | 15383700 |
|  | 15385519 |
|  | 15388652 |
|  | 15389560 |
|  | 15452110 |
|  | 15467358 |
|  | 15485831 |
|  | 15485842 |
|  | 15501798 |
|  | 15501801 |
|  | 15512964 |
|  | 15514019 |
|  | 15514106 |
|  | 15520180 |
|  | 15522205 |
|  | 1552283 |
|  | 15522996 |
|  | 1552482 |
|  | 15533287 |
|  | 15533863 |
|  | 15536133 |
|  | 15536198 |
|  | 15536567 |
|  | 15537542 |
|  | 15542662 |
|  | 15550384 |
|  | 15576826 |
|  | 15578092 |
|  | 15579454 |
|  | 15583753 |
|  | 15592751 |
|  | 15596807 |
|  | 15596827 |
|  | 15599404 |
|  | 15601669 |
|  | 15604121 |
|  | 15613243 |
|  | 15614134 |
|  | 15614439 |
|  | 15618139 |
|  | 15625085 |
|  | 15626822 |
|  | 15629131 |
|  | 15632117 |
|  | 15640147 |
|  | 15647276 |
|  | 15654515 |
|  | 15655512 |
|  | 15657949 |
|  | 15661037 |
|  | 15661932 |
|  | 15664162 |
|  | 15668736 |
|  | 15673605 |
|  | 15678118 |
|  | 15681845 |
|  | 15698414 |
|  | 15699076 |
|  | 15701678 |
|  | 15708985 |
|  | 15715082 |
|  | 15717269 |
|  | 1572101 |
|  | 15728492 |
|  | 15730385 |
|  | 15735680 |
|  | 15743782 |
|  | 15746179 |
|  | 15763541 |
|  | 15769461 |
|  | 1577194 |
|  | 15780956 |
|  | 15790681 |
|  | 15793291 |
|  | 15803053 |
|  | 15809914 |
|  | 1580993 |
|  | 15814712 |
|  | 15823270 |
|  | 15824857 |
|  | 15826936 |
|  | 15831559 |
|  | 15832287 |
|  | 15840716 |
|  | 15845526 |
|  | 15851600 |
|  | 15855353 |
|  | 15857826 |
|  | 15869941 |
|  | 15875767 |
|  | 15887111 |
|  | 15893958 |
|  | 15899409 |
|  | 15899518 |
|  | 15905096 |
|  | 15908180 |
|  | 15908387 |
|  | 15908396 |
|  | 15923312 |
|  | 15923614 |
|  | 15935078 |
|  | 15937121 |
|  | 15968000 |
|  | 15972657 |
|  | 15993497 |
|  | 15996660 |
|  | 16000198 |
|  | 16012953 |
|  | 16015367 |
|  | 1602141 |
|  | 16026578 |
|  | 16037568 |
|  | 16039576 |
|  | 16039725 |
|  | 16040991 |
|  | 16046471 |
|  | 16046706 |
|  | 16081822 |
|  | 16081883 |
|  | 16083851 |
|  | 16085647 |
|  | 1611993 |
|  | 16123045 |
|  | 16123319 |
|  | 1612651 |
|  | 16141072 |
|  | 16141073 |
|  | 16141211 |
|  | 16147974 |
|  | 16158333 |
|  | 16168373 |
|  | 16177303 |
|  | 16177310 |
|  | 16177323 |
|  | 1618860 |
|  | 16199483 |
|  | 16210605 |
|  | 16223857 |
|  | 16225850 |
|  | 16230370 |
|  | 16247001 |
|  | 16253995 |
|  | 16260493 |
|  | 16263419 |
|  | 16282134 |
|  | 16285886 |
|  | 16287858 |
|  | 16294221 |
|  | 16294222 |
|  | 16297636 |
|  | 16314479 |
|  | 16317093 |
|  | 16322943 |
|  | 16339842 |
|  | 16362898 |
|  | 16373342 |
|  | 16385082 |
|  | 16402554 |
|  | 16406654 |
|  | 16423921 |
|  | 16427608 |
|  | 16428767 |
|  | 16428769 |
|  | 16444258 |
|  | 16444265 |
|  | 16446372 |
|  | 16469053 |
|  | 16507891 |
|  | 16508014 |
|  | 16516847 |
|  | 16517114 |
|  | 16517600 |
|  | 16546488 |
|  | 16547261 |
|  | 16547515 |
|  | 16554296 |
|  | 16569679 |
|  | 16581975 |
|  | 16595132 |
|  | 16601113 |
|  | 16616573 |
|  | 16618605 |
|  | 16670337 |
|  | 16670341 |
|  | 16675448 |
|  | 16696900 |
|  | 16698931 |
|  | 16702408 |
|  | 16705149 |
|  | 16705172 |
|  | 16709824 |
|  | 16714545 |
|  | 16714568 |
|  | 16720574 |
|  | 16723700 |
|  | 1673964 |
|  | 16741956 |
|  | 16759646 |
|  | 1676977 |
|  | 16785562 |
|  | 16790355 |
|  | 16790816 |
|  | 16807245 |
|  | 16808326 |
|  | 16828290 |
|  | 16831216 |
|  | 16835229 |
|  | 16837794 |
|  | 16856209 |
|  | 16861641 |
|  | 16863996 |
|  | 1686413 |
|  | 16875741 |
|  | 16893457 |
|  | 16908863 |
|  | 16914507 |
|  | 16927297 |
|  | 16940526 |
|  | 16947419 |
|  | 16956609 |
|  | 16966414 |
|  | 16978451 |
|  | 16983719 |
|  | 16985520 |
|  | 17003115 |
|  | 17010424 |
|  | 17015694 |
|  | 17030606 |
|  | 17035338 |
|  | 17041003 |
|  | 17055451 |
|  | 17065459 |
|  | 17071594 |
|  | 17075771 |
|  | 17079668 |
|  | 17085481 |
|  | 17101606 |
|  | 17127813 |
|  | 17151265 |
|  | 17200442 |
|  | 17243316 |
|  | 1740661 |
|  | 1794058 |
|  | 1975480 |
|  | 1978715 |
|  | 2179951 |
|  | 2228046 |
|  | 2249881 |
|  | 2272660 |
|  | 2351934 |
|  | 2419912 |
|  | 2452194 |
|  | 2509559 |
|  | 2574145 |
|  | 2818653 |
|  | 2836146 |
|  | 2885372 |
|  | 2989794 |
|  | 3010124 |
|  | 3024123 |
|  | 3027565 |
|  | 3040015 |
|  | 3110291 |
|  | 3490653 |
|  | 3684584 |
|  | 3898078 |
|  | 6840816 |
|  | 7507352 |
|  | 7507449 |
|  | 7512527 |
|  | 7513199 |
|  | 7513301 |
|  | 7515767 |
|  | 7521373 |
|  | 7522842 |
|  | 7527452 |
|  | 7532178 |
|  | 7540941 |
|  | 7542280 |
|  | 7544321 |
|  | 7544870 |
|  | 7558022 |
|  | 7560085 |
|  | 7561052 |
|  | 7566090 |
|  | 7579385 |
|  | 7579389 |
|  | 7583354 |
|  | 7586747 |
|  | 7606797 |
|  | 7608544 |
|  | 7615010 |
|  | 7624137 |
|  | 7628389 |
|  | 7629174 |
|  | 7636205 |
|  | 7636227 |
|  | 7636260 |
|  | 7642536 |
|  | 7642621 |
|  | 7650392 |
|  | 7693816 |
|  | 7724598 |
|  | 7731137 |
|  | 7735226 |
|  | 7738914 |
|  | 7768593 |
|  | 7799917 |
|  | 7813805 |
|  | 7814894 |
|  | 7822027 |
|  | 7826951 |
|  | 7842491 |
|  | 7846035 |
|  | 7860994 |
|  | 7873786 |
|  | 7882592 |
|  | 7890367 |
|  | 7916002 |
|  | 7919662 |
|  | 7927491 |
|  | 7928185 |
|  | 7929839 |
|  | 7930875 |
|  | 7931089 |
|  | 7935058 |
|  | 7937857 |
|  | 7947998 |
|  | 7948748 |
|  | 7957572 |
|  | 7963536 |
|  | 7963539 |
|  | 7964480 |
|  | 7972071 |
|  | 7974484 |
|  | 7988471 |
|  | 7995944 |
|  | 7996051 |
|  | 8005666 |
|  | 8011213 |
|  | 8039244 |
|  | 8039834 |
|  | 8046244 |
|  | 8051101 |
|  | 8060345 |
|  | 8064245 |
|  | 8080166 |
|  | 8084607 |
|  | 8088324 |
|  | 8097205 |
|  | 8100792 |
|  | 8118867 |
|  | 8119415 |
|  | 8119735 |
|  | 8132347 |
|  | 8138038 |
|  | 8139551 |
|  | 8148154 |
|  | 8157957 |
|  | 8169397 |
|  | 8169531 |
|  | 8171316 |
|  | 8171322 |
|  | 8176222 |
|  | 8182150 |
|  | 8197147 |
|  | 8207244 |
|  | 8207246 |
|  | 8207651 |
|  | 8208546 |
|  | 8228806 |
|  | 8231109 |
|  | 8242763 |
|  | 8243279 |
|  | 8275935 |
|  | 8275942 |
|  | 8288770 |
|  | 8319585 |
|  | 8339588 |
|  | 8360023 |
|  | 8387892 |
|  | 8387893 |
|  | 8395024 |
|  | 8399834 |
|  | 8409382 |
|  | 8409521 |
|  | 8418212 |
|  | 8464030 |
|  | 8485234 |
|  | 8491516 |
|  | 8504748 |
|  | 8529837 |
|  | 8537128 |
|  | 8550204 |
|  | 8554587 |
|  | 8568256 |
|  | 8607873 |
|  | 8609993 |
|  | 8617220 |
|  | 8622125 |
|  | 8635284 |
|  | 8639904 |
|  | 8642347 |
|  | 8647916 |
|  | 8650181 |
|  | 8662753 |
|  | 8662965 |
|  | 8662983 |
|  | 8671586 |
|  | 8675207 |
|  | 8686743 |
|  | 8691140 |
|  | 8699818 |
|  | 8724002 |
|  | 8754800 |
|  | 8757625 |
|  | 8757945 |
|  | 8770556 |
|  | 8778035 |
|  | 8781564 |
|  | 8786318 |
|  | 8791485 |
|  | 8811936 |
|  | 8812413 |
|  | 8823306 |
|  | 8835376 |
|  | 8838580 |
|  | 8858135 |
|  | 8864118 |
|  | 8864119 |
|  | 8864120 |
|  | 8867667 |
|  | 8870704 |
|  | 8879212 |
|  | 8881659 |
|  | 8892621 |
|  | 8901048 |
|  | 8911153 |
|  | 8912006 |
|  | 8912010 |
|  | 8919359 |
|  | 8943375 |
|  | 8967504 |
|  | 9006341 |
|  | 9021146 |
|  | 9022125 |
|  | 9024304 |
|  | 9033272 |
|  | 9034190 |
|  | 9034191 |
|  | 9036957 |
|  | 9037072 |
|  | 9058758 |
|  | 9089109 |
|  | 9093874 |
|  | 9099747 |
|  | 9130643 |
|  | 9130661 |
|  | 9144505 |
|  | 9164935 |
|  | 9166104 |
|  | 9169725 |
|  | 9169855 |
|  | 9177215 |
|  | 9182689 |
|  | 9196000 |
|  | 9202217 |
|  | 9223320 |
|  | 9233639 |
|  | 9247586 |
|  | 9256477 |
|  | 9287059 |
|  | 9305915 |
|  | 9307073 |
|  | 9314845 |
|  | 9335502 |
|  | 9344502 |
|  | 9348316 |
|  | 9353071 |
|  | 9358756 |
|  | 9364332 |
|  | 9366433 |
|  | 9367897 |
|  | 9368614 |
|  | 9368616 |
|  | 9372929 |
|  | 9389742 |
|  | 9390635 |
|  | 9390694 |
|  | 9396768 |
|  | 9398854 |
|  | 9426403 |
|  | 9427610 |
|  | 9464804 |
|  | 9500515 |
|  | 9507200 |
|  | 9521083 |
|  | 9540968 |
|  | 9565639 |
|  | 9565643 |
|  | 9688988 |
|  | 9716651 |
|  | 9736029 |
|  | 9763613 |
|  | 9782127 |
|  | 9789258 |
|  | 9796913 |
|  | 9802973 |
|  | 9832509 |
|  | 9834074 |
|  | 9839161 |
|  | 9843922 |
|  | 9846494 |
|  | 9854025 |
|  | 9862349 |
|  | 9874578 |
|  | 9878080 |
|  | 9886396 |
|  | 9892616 |
|  | 9892622 |
|  | 9894568 |
|  | 9916692 |
|  | 9916727 |
|  | 9918534 |
|  | 9933106 |
|  | 10339575 |
|  | 10369255 |
|  | 10376933 |
|  | 10438928 |
|  | 10450718 |
|  | 10490959 |
|  | 10669763 |
|  | 10747938 |
|  | 10748004 |
|  | 10775602 |
|  | 10799478 |
|  | 10820279 |
|  | 10839541 |
|  | 10843712 |
|  | 10929056 |
|  | 10964543 |
|  | 11273064 |
|  | 11423177 |
|  | 11500363 |
|  | 11522182 |
|  | 11579140 |
|  | 11689614 |
|  | 11697432 |
|  | 11724772 |
|  | 11725485 |
|  | 11730934 |
|  | 11744409 |
|  | 11751963 |
|  | 11774563 |
|  | 11774822 |
|  | 11776328 |
|  | 11781187 |
|  | 11792123 |
|  | 11798770 |
|  | 11799112 |
|  | 11801679 |
|  | 11816715 |
|  | 11831440 |
|  | 11831864 |
|  | 11838837 |
|  | 11841482 |
|  | 11841484 |
|  | 11841486 |
|  | 11846846 |
|  | 11849390 |
|  | 11849463 |
|  | 11857057 |
|  | 11858187 |
|  | 11860474 |
|  | 11861275 |
|  | 11864973 |
|  | 11868623 |
|  | 11874184 |
|  | 11874815 |
|  | 11877397 |
|  | 11884401 |
|  | 11891016 |
|  | 11891022 |
|  | 11896460 |
|  | 11904678 |
|  | 11907116 |
|  | 11909956 |
|  | 11911478 |
|  | 11916086 |
|  | 11920321 |
|  | 11924527 |
|  | 11940577 |
|  | 11954826 |
|  | 11958572 |
|  | 11960393 |
|  | 11962732 |
|  | 11966578 |
|  | 11968047 |
|  | 11968052 |
|  | 11971816 |
|  | 11971973 |
|  | 11975986 |
|  | 11979305 |
|  | 11980617 |
|  | 11983108 |
|  | 11991675 |
|  | 12004062 |
|  | 12006640 |
|  | 12010662 |
|  | 12011375 |
|  | 12019209 |
|  | 12021142 |
|  | 12030367 |
|  | 12031914 |
|  | 12032749 |
|  | 12032830 |
|  | 12034045 |
|  | 12036088 |
|  | 12039524 |
|  | 12042033 |
|  | 12048203 |
|  | 12050189 |
|  | 12054739 |
|  | 12055326 |
|  | 12058258 |
|  | 12067299 |
|  | 12067756 |
|  | 12078789 |
|  | 12084660 |
|  | 12086951 |
|  | 12086962 |
|  | 12089610 |
|  | 12090473 |
|  | 12091169 |
|  | 12091338 |
|  | 12091490 |
|  | 12097398 |
|  | 12100025 |
|  | 12101072 |
|  | 12101079 |
|  | 12105273 |
|  | 12107724 |
|  | 12115605 |
|  | 12115625 |
|  | 12116197 |
|  | 12117921 |
|  | 12117943 |
|  | 12117953 |
|  | 12133434 |
|  | 12135128 |
|  | 12135369 |
|  | 12135759 |
|  | 12144133 |
|  | 12145682 |
|  | 12148593 |
|  | 12149604 |
|  | 12153747 |
|  | 12160518 |
|  | 12161098 |
|  | 12161427 |
|  | 12162435 |
|  | 12162437 |
|  | 12162871 |
|  | 12163195 |
|  | 12163370 |
|  | 12165487 |
|  | 12165799 |
|  | 12167698 |
|  | 12170268 |
|  | 12171600 |
|  | 12183057 |
|  | 12187026 |
|  | 12192111 |
|  | 12195699 |
|  | 12198621 |
|  | 12200397 |
|  | 12202945 |
|  | 12205044 |
|  | 12213594 |
|  | 12230503 |
|  | 12237295 |
|  | 12240899 |
|  | 12244188 |
|  | 12270556 |
|  | 12296856 |
|  | 12297049 |
|  | 12297050 |
|  | 12297113 |
|  | 12297724 |
|  | 12368210 |
|  | 12370499 |
|  | 12378118 |
|  | 12383455 |
|  | 12387878 |
|  | 12387879 |
|  | 12391248 |
|  | 12391840 |
|  | 12392301 |
|  | 12393608 |
|  | 12393619 |
|  | 12393755 |
|  | 12396374 |
|  | 12396377 |
|  | 12396555 |
|  | 12397597 |
|  | 12406900 |
|  | 12420104 |
|  | 12423676 |
|  | 12424537 |
|  | 12438348 |
|  | 12438370 |
|  | 12438440 |
|  | 12441140 |
|  | 12441346 |
|  | 12444137 |
|  | 12445207 |
|  | 12445803 |
|  | 12447749 |
|  | 12457980 |
|  | 12459320 |
|  | 12482669 |
|  | 12485196 |
|  | 12485861 |
|  | 12496175 |
|  | 12496396 |
|  | 12501066 |
|  | 12506026 |
|  | 12508775 |
|  | 12528108 |
|  | 12530117 |
|  | 12530118 |
|  | 12538692 |
|  | 12542496 |
|  | 12548511 |
|  | 12553033 |
|  | 12555203 |
|  | 12574344 |
|  | 12574959 |
|  | 12580317 |
|  | 12581498 |
|  | 12587980 |
|  | 12591926 |
|  | 12603009 |
|  | 12606947 |
|  | 12609526 |
|  | 12620429 |
|  | 12622777 |
|  | 12626032 |
|  | 12631070 |
|  | 12632436 |
|  | 12637641 |
|  | 12640117 |
|  | 12644006 |
|  | 12644590 |
|  | 12645577 |
|  | 12655064 |
|  | 12663241 |
|  | 12673844 |
|  | 12679941 |
|  | 12690081 |
|  | 12692009 |
|  | 12692291 |
|  | 12694213 |
|  | 12699902 |
|  | 12702205 |
|  | 12707271 |
|  | 12707358 |
|  | 12709814 |
|  | 12714268 |
|  | 12714269 |
|  | 12714560 |
|  | 12715715 |
|  | 12720537 |
|  | 12730860 |
|  | 12744771 |
|  | 12746914 |
|  | 12747595 |
|  | 12750172 |
|  | 12751024 |
|  | 12753658 |
|  | 12762136 |
|  | 12768019 |
|  | 12773519 |
|  | 12783210 |
|  | 12787424 |
|  | 12788886 |
|  | 1279199 |
|  | 12792476 |
|  | 12794527 |
|  | 12795334 |
|  | 12795791 |
|  | 12799216 |
|  | 12802423 |
|  | 12802709 |
|  | 12804035 |
|  | 12810683 |
|  | 12811828 |
|  | 12815949 |
|  | 12823767 |
|  | 12824709 |
|  | 12826369 |
|  | 12829646 |
|  | 12829659 |
|  | 12837287 |
|  | 12839942 |
|  | 12849705 |
|  | 12850624 |
|  | 12850808 |
|  | 12850811 |
|  | 12851716 |
|  | 12855762 |
|  | 12859597 |
|  | 12864989 |
|  | 12867430 |
|  | 12869351 |
|  | 12870175 |
|  | 12871882 |
|  | 12876073 |
|  | 12878172 |
|  | 12880679 |
|  | 12882963 |
|  | 12888880 |
|  | 12898475 |
|  | 12915457 |
|  | 12923493 |
|  | 12925536 |
|  | 12928370 |
|  | 12930304 |
|  | 12934185 |
|  | 12940515 |
|  | 12952969 |
|  | 12953135 |
|  | 12954601 |
|  | 12957651 |
|  | 12959221 |
|  | 12959973 |
|  | 12960156 |
|  | 12960255 |
|  | 12960355 |
|  | 12960751 |
|  | 12966432 |
|  | 12967650 |
|  | 12969378 |
|  | 12970400 |
|  | 1331108 |
|  | 1402651 |
|  | 14500462 |
|  | 14500687 |
|  | 14514692 |
|  | 14519430 |
|  | 14519761 |
|  | 14530204 |
|  | 14530285 |
|  | 14530313 |
|  | 14530362 |
|  | 14530365 |
|  | 14532277 |
|  | 14534724 |
|  | 14550782 |
|  | 14555463 |
|  | 14572449 |
|  | 14572618 |
|  | 14581470 |
|  | 14592496 |
|  | 14593215 |
|  | 14606512 |
|  | 14607690 |
|  | 14607966 |
|  | 14612430 |
|  | 14615042 |
|  | 14623370 |
|  | 14625134 |
|  | 14630568 |
|  | 14637275 |
|  | 14644777 |
|  | 14652008 |
|  | 14659806 |
|  | 14662866 |
|  | 14662883 |
|  | 14671137 |
|  | 14681231 |
|  | 14681891 |
|  | 14685701 |
|  | 14688105 |
|  | 14708348 |
|  | 14714556 |
|  | 14715628 |
|  | 14718619 |
|  | 14727453 |
|  | 14731353 |
|  | 14732222 |
|  | 14732344 |
|  | 14734776 |
|  | 14734780 |
|  | 14741380 |
|  | 14743216 |
|  | 14744790 |
|  | 14756669 |
|  | 14758530 |
|  | 14760938 |
|  | 14761884 |
|  | 14767724 |
|  | 14962095 |
|  | 14963043 |
|  | 14967554 |
|  | 14978035 |
|  | 14978132 |
|  | 14979058 |
|  | 14988320 |
|  | 14990112 |
|  | 14992821 |
|  | 15003925 |
|  | 15009105 |
|  | 15009176 |
|  | 15013759 |
|  | 15016407 |
|  | 15019283 |
|  | 15027477 |
|  | 15036247 |
|  | 15036607 |
|  | 15041705 |
|  | 15043686 |
|  | 15044327 |
|  | 15055475 |
|  | 15055529 |
|  | 15057268 |
|  | 15063602 |
|  | 15066699 |
|  | 15068388 |
|  | 15070815 |
|  | 15071492 |
|  | 15086488 |
|  | 15104370 |
|  | 15104672 |
|  | 15124210 |
|  | 15124762 |
|  | 15128741 |
|  | 15128916 |
|  | 15131465 |
|  | 15140018 |
|  | 15142990 |
|  | 15145213 |
|  | 15145954 |
|  | 15146559 |
|  | 15152368 |
|  | 15157159 |
|  | 15158680 |
|  | 15167972 |
|  | 15182332 |
|  | 15188512 |
|  | 15191888 |
|  | 15194284 |
|  | 15202783 |
|  | 15203561 |
|  | 15212671 |
|  | 15221977 |
|  | 15223607 |
|  | 15223612 |
|  | 15223990 |
|  | 15230290 |
|  | 15232637 |
|  | 15238257 |
|  | 15239085 |
|  | 15251463 |
|  | 15254878 |
|  | 15256287 |
|  | 15257049 |
|  | 15269840 |
|  | 15271933 |
|  | 15273737 |
|  | 15277733 |
|  | 15280021 |
|  | 15284722 |
|  | 15288471 |
|  | 15304089 |
|  | 15308875 |
|  | 15313687 |
|  | 15315967 |
|  | 15316669 |
|  | 15319381 |
|  | 15326480 |
|  | 15327404 |
|  | 15331607 |
|  | 15332393 |
|  | 15340354 |
|  | 15345185 |
|  | 15358608 |
|  | 15374002 |
|  | 15381184 |
|  | 15388258 |
|  | 15388264 |
|  | 15449074 |
|  | 15458430 |
|  | 15469436 |
|  | 15474092 |
|  | 15479128 |
|  | 15482484 |
|  | 15485837 |
|  | 15486341 |
|  | 15489542 |
|  | 15492857 |
|  | 15495787 |
|  | 15504546 |
|  | 15504978 |
|  | 15507966 |
|  | 15507967 |
|  | 1550865 |
|  | 15515013 |
|  | 15516323 |
|  | 15523649 |
|  | 15523691 |
|  | 15524285 |
|  | 15531184 |
|  | 15537425 |
|  | 15540224 |
|  | 15542843 |
|  | 15543003 |
|  | 15545623 |
|  | 15545823 |
|  | 15546338 |
|  | 15555625 |
|  | 15556647 |
|  | 15560757 |
|  | 15564333 |
|  | 15572357 |
|  | 15583831 |
|  | 15587751 |
|  | 15592496 |
|  | 15597152 |
|  | 15600254 |
|  | 15604419 |
|  | 15611081 |
|  | 15619425 |
|  | 15620462 |
|  | 15626980 |
|  | 15638425 |
|  | 15640148 |
|  | 15641051 |
|  | 15641080 |
|  | 15649304 |
|  | 15650331 |
|  | 15652446 |
|  | 15654990 |
|  | 15660126 |
|  | 15667911 |
|  | 15668241 |
|  | 15670770 |
|  | 15677734 |
|  | 15679580 |
|  | 15681825 |
|  | 15685549 |
|  | 15694363 |
|  | 15694386 |
|  | 15701637 |
|  | 15710474 |
|  | 15710602 |
|  | 15712606 |
|  | 15713215 |
|  | 15714129 |
|  | 15717274 |
|  | 15722350 |
|  | 15722553 |
|  | 15723199 |
|  | 15725578 |
|  | 15727562 |
|  | 15728381 |
|  | 15731048 |
|  | 15754412 |
|  | 15755722 |
|  | 15766528 |
|  | 15766560 |
|  | 15777836 |
|  | 15780075 |
|  | 15780879 |
|  | 15784408 |
|  | 15784410 |
|  | 15788755 |
|  | 15797745 |
|  | 15797957 |
|  | 15798948 |
|  | 15800781 |
|  | 15803022 |
|  | 15809899 |
|  | 15810887 |
|  | 15816837 |
|  | 15817702 |
|  | 15825968 |
|  | 15828019 |
|  | 15837333 |
|  | 15845475 |
|  | 15845648 |
|  | 15847100 |
|  | 15851552 |
|  | 15860935 |
|  | 15866693 |
|  | 15870936 |
|  | 15878915 |
|  | 15879291 |
|  | 15885323 |
|  | 15895461 |
|  | 15896304 |
|  | 15901240 |
|  | 15902922 |
|  | 15904673 |
|  | 15919211 |
|  | 15923644 |
|  | 15927855 |
|  | 15929757 |
|  | 15931230 |
|  | 15935956 |
|  | 15951664 |
|  | 15955385 |
|  | 15956282 |
|  | 15956727 |
|  | 15963988 |
|  | 15964333 |
|  | 15967797 |
|  | 15968724 |
|  | 15971421 |
|  | 15972386 |
|  | 15978325 |
|  | 15979578 |
|  | 15979955 |
|  | 15984988 |
|  | 15985479 |
|  | 15987860 |
|  | 15988006 |
|  | 15990085 |
|  | 15993097 |
|  | 15994321 |
|  | 16004996 |
|  | 16006475 |
|  | 16009345 |
|  | 16011481 |
|  | 16024973 |
|  | 16027740 |
|  | 16029496 |
|  | 16039994 |
|  | 16040075 |
|  | 16051302 |
|  | 16082153 |
|  | 16087126 |
|  | 16105652 |
|  | 16105945 |
|  | 16107336 |
|  | 16109906 |
|  | 16115611 |
|  | 16134057 |
|  | 16135957 |
|  | 16135983 |
|  | 16137184 |
|  | 16140265 |
|  | 16143422 |
|  | 16148157 |
|  | 16170833 |
|  | 16176285 |
|  | 16181755 |
|  | 16199883 |
|  | 16202847 |
|  | 16203766 |
|  | 16207331 |
|  | 16210389 |
|  | 16211257 |
|  | 16215460 |
|  | 16224057 |
|  | 16224188 |
|  | 16227624 |
|  | 16239914 |
|  | 16243340 |
|  | 16246225 |
|  | 16246259 |
|  | 16251478 |
|  | 16252073 |
|  | 16253303 |
|  | 16260352 |
|  | 16260427 |
|  | 16260783 |
|  | 16269960 |
|  | 16272296 |
|  | 16276011 |
|  | 16280327 |
|  | 16286275 |
|  | 16314440 |
|  | 16318585 |
|  | 16319659 |
|  | 16325265 |
|  | 16331275 |
|  | 16339571 |
|  | 16339574 |
|  | 16343435 |
|  | 16359550 |
|  | 16368349 |
|  | 16386594 |
|  | 16393214 |
|  | 16397758 |
|  | 16407614 |
|  | 16410066 |
|  | 16410254 |
|  | 16413198 |
|  | 16418737 |
|  | 16423623 |
|  | 16424170 |
|  | 16426238 |
|  | 16427740 |
|  | 16427798 |
|  | 16428794 |
|  | 16431125 |
|  | 16458016 |
|  | 16467041 |
|  | 16474009 |
|  | 16475830 |
|  | 16476505 |
|  | 16503400 |
|  | 16505113 |
|  | 16507146 |
|  | 16516271 |
|  | 16518755 |
|  | 16519772 |
|  | 16533817 |
|  | 16543241 |
|  | 16545788 |
|  | 16549165 |
|  | 16552356 |
|  | 16554734 |
|  | 16564576 |
|  | 16564703 |
|  | 16567828 |
|  | 16569263 |
|  | 16571778 |
|  | 16574427 |
|  | 16580225 |
|  | 16585124 |
|  | 16596274 |
|  | 16596276 |
|  | 16611251 |
|  | 16617094 |
|  | 16622779 |
|  | 16628468 |
|  | 16633737 |
|  | 16647242 |
|  | 16698276 |
|  | 16699450 |
|  | 16699452 |
|  | 16707559 |
|  | 16716376 |
|  | 16718633 |
|  | 16720051 |
|  | 16723255 |
|  | 16723520 |
|  | 16728705 |
|  | 16751383 |
|  | 16757480 |
|  | 16778150 |
|  | 16785565 |
|  | 16796744 |
|  | 16798739 |
|  | 16804395 |
|  | 16824070 |
|  | 16827183 |
|  | 16827185 |
|  | 16829620 |
|  | 16835238 |
|  | 16857678 |
|  | 16861224 |
|  | 16864906 |
|  | 16865291 |
|  | 16879054 |
|  | 16879221 |
|  | 16885196 |
|  | 16891799 |
|  | 16895750 |
|  | 16895791 |
|  | 16896069 |
|  | 16908592 |
|  | 16908746 |
|  | 16919469 |
|  | 16919536 |
|  | 16931032 |
|  | 16935932 |
|  | 16936264 |
|  | 16938460 |
|  | 16951360 |
|  | 16952484 |
|  | 16952987 |
|  | 16960936 |
|  | 16982833 |
|  | 16984517 |
|  | 16987073 |
|  | 16987811 |
|  | 16998242 |
|  | 17003486 |
|  | 17006329 |
|  | 17007883 |
|  | 17016618 |
|  | 17034639 |
|  | 17052690 |
|  | 17054908 |
|  | 17055360 |
|  | 17064665 |
|  | 17069807 |
|  | 17071494 |
|  | 17074928 |
|  | 17079667 |
|  | 17082635 |
|  | 17084384 |
|  | 17097497 |
|  | 17105909 |
|  | 17109502 |
|  | 17117950 |
|  | 17132473 |
|  | 2158863 |
|  | 2413547 |
|  | 2537468 |
|  | 2848815 |
|  | 2995927 |
|  | 3001529 |
|  | 3031624 |
|  | 3464978 |
|  | 3552045 |
|  | 3555974 |
|  | 3856324 |
|  | 3883195 |
|  | 3932069 |
|  | 6392892 |
|  | 7494249 |
|  | 7523444 |
|  | 7526541 |
|  | 7693046 |
|  | 7695626 |
|  | 7818827 |
|  | 7859743 |
|  | 7935762 |
|  | 8336080 |
|  | 8590321 |
|  | 8597870 |
|  | 8724035 |
|  | 8852605 |
|  | 9110146 |
|  | 9269771 |
|  | 9278385 |
|  | 9334723 |
|  | 9378998 |
|  | 9438495 |
|  | 9449720 |
|  | 9552007 |
|  | 9639071 |
|  | 9651389 |
|  | 9670843 |
|  | 9730685 |
|  | 9828130 |
|  | 10202016 |
|  | 10205166 |
|  | 14656967 |
|  | 15489334 |
|  | 16133992 |
|  | 16473522 |
|  | 16707968 |
|  | 16730227 |
|  | 16814297 |
|  | 16820934 |
|  | 16859780 |
|  | 16897113 |
|  | 16916598 |
|  | 16930761 |
|  | 16953332 |
|  | 16980557 |
|  | 16988194 |
|  | 17014432 |
|  | 17057944 |
|  | 17063315 |
|  | 17087739 |
|  | 17090647 |
|  | 17101499 |
|  | 17114809 |
|  | 17133355 |
|  | 17141882 |
|  | 17143972 |
|  | 17164537 |
|  | 17166738 |
|  | 17171665 |
|  | 17185628 |
|  | 17192953 |
|  | 17194482 |
|  | 17208210 |
|  | 17208287 |
|  | 17208993 |
|  | 17209781 |
|  | 17213876 |
|  | 17216494 |
|  | 17221211 |
|  | 17227768 |
|  | 17234183 |
|  | 17234379 |
|  | 17238853 |
|  | 17268200 |
|  | 17272816 |
|  | 17277048 |
|  | 17277102 |
|  | 17298451 |
|  | 17298885 |
|  | 17303559 |
|  | 17304101 |
|  | 17304103 |
|  | 17305696 |
|  | 17307728 |
|  | 17328044 |
|  | 17330135 |
|  | 17335072 |
|  | 17343250 |
|  | 17346438 |
|  | 17347550 |
|  | 17349210 |
|  | 17356374 |
|  | 17356550 |
|  | 17359518 |
|  | 17373637 |
|  | 17380115 |
|  | 17382880 |
|  | 17389591 |
|  | 17395331 |
|  | 17397790 |
|  | 17397798 |
|  | 17397847 |
|  | 17401435 |
|  | 17405915 |
|  | 17408448 |
|  | 17412556 |
|  | 17416344 |
|  | 17426528 |
|  | 17428673 |
|  | 17431682 |
|  | 17434489 |
|  | 17439892 |
|  | 17442953 |
|  | 17445540 |
|  | 17449468 |
|  | 17450233 |
|  | 17452160 |
|  | 17452460 |
|  | 17452777 |
|  | 17454170 |
|  | 17458901 |
|  | 17460948 |
|  | 17475896 |
|  | 17477779 |
|  | 17479109 |
|  | 17482757 |
|  | 17483581 |
|  | 17493155 |
|  | 17493863 |
|  | 17495957 |
|  | 17498265 |
|  | 17498268 |
|  | 17499233 |
|  | 17505301 |
|  | 17509552 |
|  | 17513605 |
|  | 17515611 |
|  | 17516296 |
|  | 17530646 |
|  | 17530706 |
|  | 17532302 |
|  | 17543146 |
|  | 17550423 |
|  | 17560079 |
|  | 17562931 |
|  | 17564320 |
|  | 17579071 |
|  | 17583675 |
|  | 17587429 |
|  | 17589947 |
|  | 17592115 |
|  | 17592300 |
|  | 17592954 |
|  | 17593951 |
|  | 17597660 |
|  | 17599062 |
|  | 17599736 |
|  | 17600229 |
|  | 17624216 |
|  | 17631138 |
|  | 17631734 |
|  | 17631742 |
|  | 17635596 |
|  | 17635597 |
|  | 17641010 |
|  | 17657677 |
|  | 17665457 |
|  | 17674321 |
|  | 17785317 |
|  | 1964681 |
|  | 2009860 |
|  | 2551905 |
|  | 2922050 |
|  | 8499947 |
|  | 9442056 |
|  | 9488135 |
|  | 10089307 |
|  | 10411942 |
|  | 10415052 |
|  | 10515827 |
|  | 10799877 |
|  | 10878371 |
|  | 10914503 |
|  | 11005868 |
|  | 11298355 |
|  | 12093867 |
|  | 12471131 |
|  | 12551985 |
|  | 12771125 |
|  | 12855817 |
|  | 12872135 |
|  | 14691292 |
|  | 14764707 |
|  | 15111301 |
|  | 15207270 |
|  | 16054986 |
|  | 16129814 |
|  | 16210607 |
|  | 16237096 |
|  | 16275384 |
|  | 16285964 |
|  | 16751399 |
|  | 16923852 |
|  | 16935850 |
|  | 16958667 |
|  | 17015619 |
|  | 17046971 |
|  | 17095513 |
|  | 17098824 |
|  | 17101148 |
|  | 17107717 |
|  | 17123788 |
|  | 17151142 |
|  | 17171644 |
|  | 17198884 |
|  | 17202326 |
|  | 17203472 |
|  | 17210619 |
|  | 17218256 |
|  | 17222972 |
|  | 17228359 |
|  | 17229673 |
|  | 17234424 |
|  | 17236235 |
|  | 17237440 |
|  | 17242187 |
|  | 17266742 |
|  | 17276429 |
|  | 17277081 |
|  | 17301687 |
|  | 17301950 |
|  | 17304113 |
|  | 17307724 |
|  | 17336618 |
|  | 17341609 |
|  | 17353445 |
|  | 17360740 |
|  | 17361131 |
|  | 17372191 |
|  | 17379643 |
|  | 17380205 |
|  | 17384464 |
|  | 17391979 |
|  | 17395890 |
|  | 17409491 |
|  | 17416608 |
|  | 17416740 |
|  | 17416966 |
|  | 17428547 |
|  | 17436232 |
|  | 17438029 |
|  | 17449582 |
|  | 17451964 |
|  | 17464333 |
|  | 17467668 |
|  | 17467814 |
|  | 17475837 |
|  | 17482668 |
|  | 17485113 |
|  | 17485464 |
|  | 17485824 |
|  | 17487773 |
|  | 17500068 |
|  | 17512902 |
|  | 17513720 |
|  | 17513784 |
|  | 17517868 |
|  | 17537727 |
|  | 17543594 |
|  | 17548042 |
|  | 17572011 |
|  | 17576366 |
|  | 17579033 |
|  | 17591872 |
|  | 17606866 |
|  | 17618911 |
|  | 17638882 |
|  | 17640882 |
|  | 17692539 |
|  | 17713535 |
|  | 2268312 |
|  | 2777790 |
|  | 3349526 |
|  | 8039702 |
|  | 9551933 |
|  | 9874572 |
|  | 15060004 |
|  | 15935297 |
|  | 16705482 |
|  | 17158358 |
|  | 17161388 |
|  | 17166735 |
|  | 17170233 |
|  | 17170234 |
|  | 17278014 |
|  | 17336456 |
|  | 17337591 |
|  | 17346684 |
|  | 17380194 |
|  | 17405916 |
|  | 17416605 |
|  | 17433709 |
|  | 17531209 |
|  | 17582939 |
|  | 17592532 |
|  | 17659374 |

---

|  |  |
| --- | --- |
| Organism | Homo sapiens {Organism urn:agi-taxid:9606} |
|  | Mus musculus {Organism urn:agi-taxid:10090} |
|  | Rattus norvegicus {Organism urn:agi-taxid:10116} |
|  | Homo sapiens |
|  | Mus musculus |
|  | Rattus norvegicus |

---

|  |  |
| --- | --- |
| Mouse chromosome position | 17 18.59 cM |
|  | 17 19.06 cM |

---

|  |  |
| --- | --- |
| OMIM ID | 191160 |
|  | 600807 |
|  | 157300 |
|  | 611162 |
|  | 248310 |

---

|  |  |
| --- | --- |
| Rat chromosome position | 20p12 |

---

|  |  |
| --- | --- |
| Hugo ID | 11892 |
|  | HGNC:11892 |

---

|  |  |
| --- | --- |
| Human chromosome position | 6p21.33 |
|  | 6p21.3 |

---

|  |  |
| --- | --- |
| Swiss-Prot Accession | P01375 |
|  | Q5STB3 |
|  | P01375.1 |
|  | A0A0R4J210 |
|  | P06804 |
|  | Q3U593 |
|  | P06804.2 |
|  | P16599 |
|  | P16599.1 |
|  | O43647 |
|  | Q9P1Q2 |
|  | Q9UIV3 |
|  | O35853 |
|  | Q62326 |
|  | Q91VF3 |
|  | Q6EE11 |
|  | Q9JI26 |
|  | Q9JI27 |
|  | C1K3N5 |
|  | Q5RT83 |
|  | Q9UBM5 |
|  | Q0X0E6 |
|  | Q3U4I6 |
|  | Q6TDG3 |
|  | TNFA\_HUMAN |
|  | TNFA\_MOUSE |
|  | TNFA\_RAT |
|  | Q5STB3\_HUMAN |
|  | Q3U593\_MOUSE |

---

|  |  |
| --- | --- |
| PIR ID | A93585 |
|  | A22908 |
|  | JU0029 |

---

|  |  |
| --- | --- |
| GenBank ID | NC\_000006 |
|  | NM\_000594 |
|  | NP\_000585 |
|  | NT\_167244 |
|  | NT\_113891 |
|  | NT\_167245 |
|  | NT\_167246 |
|  | NT\_167247 |
|  | NT\_167248 |
|  | NT\_167249 |
|  | NG\_007462 |
|  | AB088112 |
|  | BAC54944 |
|  | AB103618 |
|  | BAF31279 |
|  | AB202113 |
|  | BAE78639 |
|  | AF129756 |
|  | AAD18091 |
|  | AJ249755 |
|  | AJ270944 |
|  | CAB65595 |
|  | AL662801 |
|  | AL662847 |
|  | AL929587 |
|  | AY066019 |
|  | AAL47581 |
|  | AY214167 |
|  | AAO21132 |
|  | AY274889 |
|  | AY274890 |
|  | AY274891 |
|  | AY274892 |
|  | AY274893 |
|  | AY274894 |
|  | AY274895 |
|  | AY274896 |
|  | AY274897 |
|  | AY274898 |
|  | AY274899 |
|  | AY274900 |
|  | AY274901 |
|  | AY274902 |
|  | AY799806 |
|  | AAV63951 |
|  | BA000025 |
|  | BAB63396 |
|  | BX248519 |
|  | BX927320 |
|  | CH471081 |
|  | EAX03424 |
|  | CR753892 |
|  | CR759886 |
|  | CR942185 |
|  | CS081473 |
|  | CAI95946 |
|  | EF471114 |
|  | EF471115 |
|  | EF540585 |
|  | EF540586 |
|  | EU338455 |
|  | KF887965 |
|  | AHJ25918 |
|  | KF887966 |
|  | AHJ25919 |
|  | KF887967 |
|  | AHJ25920 |
|  | KF887968 |
|  | AHJ25921 |
|  | KF887969 |
|  | AHJ25922 |
|  | KF887970 |
|  | AHJ25923 |
|  | KF887971 |
|  | AHJ25924 |
|  | KF887972 |
|  | AHJ25925 |
|  | KF934400 |
|  | KF934401 |
|  | KP339511 |
|  | KP339512 |
|  | AKI85808 |
|  | KX953765 |
|  | KY500676 |
|  | AQY77148 |
|  | KY500677 |
|  | AQY77149 |
|  | KY500678 |
|  | AQY77150 |
|  | LT546440 |
|  | M16441 |
|  | AAA61200 |
|  | M26331 |
|  | AAA36758 |
|  | MH287061 |
|  | QAY29141 |
|  | U42625 |
|  | AAB01765 |
|  | X02910 |
|  | CAA26669 |
|  | Y14768 |
|  | CAA75070 |
|  | Z15026 |
|  | CAA78745 |
|  | AB451492 |
|  | BAG70306 |
|  | AF043342 |
|  | AAC03542 |
|  | AF098751 |
|  | AAF71992 |
|  | AJ227911 |
|  | AJ251878 |
|  | CAB63904 |
|  | AJ251879 |
|  | CAB63905 |
|  | AK314960 |
|  | BAG37464 |
|  | BC028148 |
|  | AAH28148 |
|  | BI908079 |
|  | BP215875 |
|  | CA306559 |
|  | FJ795028 |
|  | ACO37640 |
|  | HQ201306 |
|  | ADV31546 |
|  | LN874317 |
|  | CTQ86082 |
|  | M10988 |
|  | AAA61198 |
|  | X01394 |
|  | CAA25650 |
|  | P01375 |
|  | NC\_000083 |
|  | NM\_001278601 |
|  | NP\_001265530 |
|  | NM\_013693 |
|  | NP\_038721 |
|  | NT\_039662 |
|  | NT\_187027 |
|  | NT\_187004 |
|  | AB039224 |
|  | BAB68748 |
|  | AB039226 |
|  | BAB68750 |
|  | AB039227 |
|  | BAB68751 |
|  | AB039228 |
|  | BAB68752 |
|  | AB039229 |
|  | BAB68753 |
|  | AB039230 |
|  | BAB68754 |
|  | AB039231 |
|  | BAB68755 |
|  | AB039232 |
|  | BAB68756 |
|  | AB062426 |
|  | BAB78397 |
|  | AB185894 |
|  | BAF02298 |
|  | AB185895 |
|  | BAF02299 |
|  | AB185896 |
|  | BAF02300 |
|  | AF109719 |
|  | AAC82484 |
|  | CH466666 |
|  | EDL26631 |
|  | EDL26632 |
|  | CR974444 |
|  | CU407309 |
|  | CU466548 |
|  | D84196 |
|  | BAA19512 |
|  | D84199 |
|  | BAA19513 |
|  | L22359 |
|  | L22360 |
|  | L22361 |
|  | L22362 |
|  | L22363 |
|  | L22364 |
|  | L22365 |
|  | M20155 |
|  | AAA40462 |
|  | M38296 |
|  | AAA40459 |
|  | U06950 |
|  | AAA18594 |
|  | U68414 |
|  | AAB65593 |
|  | Y00467 |
|  | CAA68530 |
|  | AK153319 |
|  | BAE31899 |
|  | AK153800 |
|  | BAE32187 |
|  | AK154223 |
|  | BAE32445 |
|  | AK155964 |
|  | BAE33525 |
|  | AY423855 |
|  | AAR00346 |
|  | BC117057 |
|  | AAI17058 |
|  | BC137720 |
|  | AAI37721 |
|  | BY167004 |
|  | LN874395 |
|  | CTQ86160 |
|  | M11731 |
|  | AAA40458 |
|  | M13049 |
|  | AAA40457 |
|  | X02611 |
|  | CAA26457 |
|  | P06804 |
|  | NC\_005119 |
|  | NM\_012675 |
|  | NP\_036807 |
|  | AC\_000088 |
|  | AAHX01099608 |
|  | AC094348 |
|  | AF329982 |
|  | AAK53568 |
|  | AF329983 |
|  | AAK53569 |
|  | AF329984 |
|  | AAK53570 |
|  | AF329985 |
|  | AAK53571 |
|  | AF329986 |
|  | AAK53572 |
|  | AF329987 |
|  | AAK53573 |
|  | AY427673 |
|  | AAR91624 |
|  | AY427674 |
|  | AAR91625 |
|  | AY427675 |
|  | AAR91626 |
|  | BX883046 |
|  | CAE84003 |
|  | CH474121 |
|  | EDL83547 |
|  | EDL83548 |
|  | D00475 |
|  | BAA00367 |
|  | L00981 |
|  | AAA16275 |
|  | L19123 |
|  | AAA42255 |
|  | AF269159 |
|  | AAF82567 |
|  | AF269160 |
|  | AAF82568 |
|  | AJ002278 |
|  | CAA05290 |
|  | BC107671 |
|  | AAI07672 |
|  | X66539 |
|  | CAA47146 |
|  | P16599 |
|  | NC\_018917 |
|  | AMYH02013452 |
|  | DQ891662 |
|  | ABM82588 |
|  | DQ894849 |
|  | ABM85775 |
|  | M35592 |
|  | AAC42098 |
|  | AC\_000039 |
|  | AAHY01132219 |
|  | AC\_000138 |
|  | ABBA01038081 |
|  | AB039225 |
|  | BAB68749 |
|  | AABR06099883 |
|  | CAI18649 |
|  | CAI41940 |
|  | CAQ09993 |
|  | CAQ06935 |
|  | CAQ07711 |
|  | NT\_007592 |
|  | NW\_001838980 |
|  | NW\_923073 |
|  | NG\_012010 |
|  | AC\_000049 |
|  | CAI18292 |
|  | CAI17678 |
|  | Q5RT83 |
|  | Q5STB3 |
|  | Q9UBM5 |
|  | NT\_039649 |
|  | NW\_001030616 |
|  | Q0X0E6 |
|  | Q3U4I6 |
|  | Q3U593 |
|  | Q6TDG3 |
|  | NW\_001084782 |
|  | NW\_047597 |
|  | NT\_113894 |
|  | CH473975 |
|  | EDL95526 |
|  | NW\_000124 |
|  | 135938 |
|  | 14091045 |
|  | 14091047 |
|  | 14091049 |
|  | 14091051 |
|  | 14091053 |
|  | 14091055 |
|  | 205254 |
|  | 220921 |
|  | 2570070 |
|  | 310232 |
|  | 395370 |
|  | 40795923 |
|  | 40795925 |
|  | 40795927 |
|  | 46237626 |
|  | 78070805 |
|  | 81886213 |
|  | 9049959 |
|  | 9049961 |
|  | Q6EE11 |
|  | 109734296 |
|  | 111036530 |
|  | 111036532 |
|  | 111036534 |
|  | 123784221 |
|  | 123796047 |
|  | 123796993 |
|  | 135935 |
|  | 15823194 |
|  | 15823196 |
|  | 15823198 |
|  | 15823200 |
|  | 15823202 |
|  | 15823204 |
|  | 15823206 |
|  | 15823208 |
|  | 15823210 |
|  | 1944348 |
|  | 1944350 |
|  | 202083 |
|  | 202085 |
|  | 202087 |
|  | 202093 |
|  | 2304957 |
|  | 37695630 |
|  | 3941741 |
|  | 495468 |
|  | 54832 |
|  | 54845 |
|  | 74142271 |
|  | 74149062 |
|  | 74178356 |
|  | 74215095 |
|  | 81870785 |
|  | 114306801 |
|  | 119623829 |
|  | 123996347 |
|  | 135934 |
|  | 15277268 |
|  | 17978883 |
|  | 20381342 |
|  | 209486 |
|  | 27544420 |
|  | 27802685 |
|  | 2905634 |
|  | 339738 |
|  | 339741 |
|  | 339764 |
|  | 37210 |
|  | 37212 |
|  | 37220 |
|  | 3805811 |
|  | 4337115 |
|  | 55740339 |
|  | 55961541 |
|  | 55961700 |
|  | 56207432 |
|  | 57209823 |
|  | 6624926 |
|  | 6624928 |
|  | 66348790 |
|  | 6688232 |
|  | 74720074 |
|  | 74727307 |
|  | 74756060 |
|  | 8090602 |
|  | 86197995 |
|  | 123981518 |
|  | 148694684 |
|  | 148694685 |
|  | 149028096 |
|  | 149028097 |

---

|  |  |
| --- | --- |
| Swiss-Prot ID | TNFA\_HUMAN |
|  | TNFA\_MOUSE |
|  | TNFA\_RAT |
|  | Q5STB3\_HUMAN |
|  | C1K3N5\_HUMAN |
|  | Q3U593\_MOUSE |

---

|  |  |
| --- | --- |
| Cell Localization | Cell membrane |
|  | Membrane |
|  | Secreted |
|  | Extracellular |

---

|  |  |
| --- | --- |
| Ensembl ID | ENSG00000232810 |
|  | ENSP00000398698.2 |
|  | ENST00000449264.3 |
|  | ENSMUSG00000024401 |
|  | ENSMUSP00000126122.1 |
|  | ENSMUST00000167924.1 |
|  | ENSMUSP00000025263.8 |
|  | ENSMUST00000025263.14 |
|  | ENSRNOG00000055156 |
|  | ENSRNOP00000074520.1 |
|  | ENSRNOT00000079677.1 |
|  | ENST00000449264.2 |
|  | ENSP00000398698 |
|  | ENST00000449264 |
|  | ENSMUSP00000126122 |
|  | ENSMUST00000167924 |
|  | ENSMUSP00000025263 |
|  | ENSMUST00000025263 |
|  | ENSRNOG00000000837 |
|  | ENSRNOP00000074520 |
|  | ENSRNOT00000079677 |
|  | ENSRNOT00000001110 |
|  | ENSRNOP00000001110 |

---

|  |  |
| --- | --- |
| MGI ID | MGI:104798 |
|  | 104798 |

---

|  |  |
| --- | --- |
| RGD ID | 3876 |

---

|  |  |
| --- | --- |
| Unigene ID | Rn.2275 |
|  | Mm.1293 |
|  | Hs.241570 |

---

|  |  |
| --- | --- |
| Homologene ID | 496 |

---

|  |  |
| --- | --- |
| Shape | Rhomb |

---

|  |  |
| --- | --- |
| IPI ID | IPI00128861 |
|  | IPI00001671 |
|  | IPI00213948 |
|  | IPI00894294 |

---

|  |  |
| --- | --- |
| Homo sapiens Chromosome position | 6p21.3 |
|  | 6 |

---

|  |  |
| --- | --- |
| Ensembl Transcript ID | ENST00000383302 |
|  | ENST00000383496 |
|  | ENST00000376122 |

---

|  |  |
| --- | --- |
| FunctionalClass | cytokine |
|  | tumor necrosis factor receptor binding |
|  | protein binding |

---

|  |  |
| --- | --- |
| Mus musculus Chromosome position | 17 19.06 cM |

---

|  |  |
| --- | --- |
| KEGG pathway | Hematopoietic cell lineage |
|  | Type II diabetes mellitus |
|  | Type I diabetes mellitus |
|  | Apoptosis |
|  | Cytokine-cytokine receptor interaction |
|  | Toll-like receptor signaling pathway |
|  | MAPK signaling pathway |
|  | TGF-beta signaling pathway |
|  | T cell receptor signaling pathway |
|  | Adipocytokine signaling pathway |
|  | Fc epsilon RI signaling pathway |
|  | Natural killer cell mediated cytotoxicity |

---

|  |  |
| --- | --- |
| Rattus norvegicus Chromosome position | 20p12 |

---

|  |  |
| --- | --- |
| Ensembl Gene ID | ENSG00000206328 |
|  | ENSG00000206439 |
|  | ENSG00000204490 |

---

|  |  |
| --- | --- |
| LocatorString | pmquery:prevacid, prilosec (omeprazole) 2002:2010[PDAT] |
|  | digtext:C:\Users\Ariadne\Customers\Elan\Elan weekly report from literature condensed.txt |
|  | pmquery:sepsis+OR+blood+poisoning+OR+Septicemia+OR+systemic+inflammatory+response+syndrome |
|  | pmquery:curcumin prostate cancer |

---
